# A systematic review of the use of bacteriophages for *in vitro* biofilm control

**DOI:** 10.1007/s10096-023-04638-1

**Published:** 2023-07-05

**Authors:** Luciana Meneses, Ana Catarina Brandão, Tom Coenye, Ana Cristina Braga, Diana Priscila Pires, Joana Azeredo

**Affiliations:** 1 CEB - Centre of Biological Engineering, University of Minho, Braga, Portugal; 2 Laboratory of Pharmaceutical Microbiology, Ghent University, Ghent, Belgium; 3 ESCMID Study Group for Biofilms (ESGB), Basel, Switzerland; 4 University of Minho, Algoritmi Center, Braga, Portugal; 5 LABBELS –Associate Laboratory, Braga, Guimarães Portugal

**Keywords:** Bacteriophages, Biofilm, *In vitro*, Systematic review

## Abstract

Bacteriophages (phages) are very promising biological agents for the prevention and control of bacterial biofilms. However, little is known about the parameters that can influence the efficacy of phages on biofilms. This systematic review provides a summary and analysis of the published data about the use of phages to control pre-formed biofilms *in vitro*, suggesting recommendations for future experiments in this area. A total of 68 articles, containing data on 605 experiments addressing the efficacy of phages to control biofilms *in vitro* were included, after a search conducted in Web of Science, Embase, and Medline (PubMed). The data collected from each experiment included information about biofilm growth conditions, phage characteristics, treatment conditions and biofilm reduction. In most cases, biofilms were formed in the surface of microtiter plates (82.5%); the median time for biofilm formation was 24 h, as is the median treatment duration. Quantification of biofilm biomass (52.6%), viable cells (25.5%) and metabolic activity (17.9%) were the most common biofilm assessment methods. Correlation analysis revealed that some phage parameters can influence the treatment outcome: higher phage concentrations were strongly associated with improved biofilm control, leading to higher levels of biofilm reduction, and phages with higher burst sizes and shorter latent periods seem to be the best candidates to control biofilms *in vitro*. However, the great variability of the methodologies used prompts the need for the development of standardized *in vitro* methodologies to characterize phage/biofilm interactions and to assess the efficacy of phages to control biofilms.

**Supplementary Information:**

The online version contains supplementary material available at 10.1007/s10096-023-04638-1.

## Introduction

The fast emergence and widespread of antibiotic-resistant bacteria constitute a global health concern that increases the need for development of new antimicrobials [1]. Bacteria have developed multiple strategies to survive in heterogeneous environments and under harsh conditions, thus becoming less susceptible to external pressures, such as antimicrobials, environmental stresses, and host immune defenses [2, 3]. One of the protective modes of bacterial growth consists in the formation of aggregates that are suspended or surface-attached and are embedded in a self-produced matrix of extracellular polymeric substances (EPS) [4, 5]. These microbial biofilms constitute the predominant form of bacterial and archaeal life, estimated to account for 40–80% of cells [6, 7].

Biofilms are widespread in diverse environmental habitats, prevailing in soil and upper oceanic sediments [6]. In clinical settings, it is estimated that biofilms are present in more than 80% of the bacterial infections of the human body [8]. The treatment of biofilm-related infections, including device-related infections and tissue-related infections, is challenging, and treatment failure is associated with recurrence of the infection [9].

Most of the difficulties experienced in the treatment of biofilm-related infections are a consequence of the high tolerance of biofilms to antibiotics [1, 9]. Therefore, there is a need of alternative strategies to effectively control these bacterial communities. A very promising approach is the use of bacteriophages, or phages, the most abundant biological entities on Earth, that specifically and exclusively infect bacterial cells [10]. Phages have been historically classified according to their morphology and the main interest for therapy has focused in three families: *Podoviridae*, *Myoviridae* and *Siphoviridae*. However, these families have been abolished in a recent taxonomy update, with the creation of the class *Caudoviricetes* comprised by new genome-based families [11]. Phages belonging to this class have important features for biofilm control. The high absolute numbers of bacteria found in biofilms and their high density and close proximity in biofilm communities allow fast replication of phages and the spread of new phage particles through the biofilm [10]. In addition, many phages produce depolymerases, which are polysaccharide-degrading enzymes used to degrade capsular polysaccharides (giving phages access to the receptors on the cell surface) or exopolysaccharides of the biofilm matrix (improving phage penetration through the biofilm) [10, 12]. Endolysins, which are phage-derived enzymes that cleave peptidoglycan, causing the lysis of bacterial cells from within, have also been found to degrade biofilms [13]. Finally, some phages can infect stationary-phase cells that are typically present in the inner layers of biofilms; these cells are destroyed when reactivated [10]. Based on these unique properties, the use of phages, phage-derived enzymes, or phages in combination with antibiotics are considered as promising strategies for biofilm control [14]. However, it is important to be aware that a complete biofilm eradication by phages is very difficult to achieve: the presence of matrix and other secreted molecules may impair phage diffusion and act as a phage decoy, the low metabolic activity of biofilm cells may impair propagation and, additionally, the presence of phage-resistant phenotypes that can proliferate during the treatment and the limited host range of phages unable to target the high diversity of the biofilm communities can be an obstacle to the success of biofilm treatment [12].

A first step towards the application of phages for biofilm control is the evaluation of their anti-biofilm activity *in vitro* [15, 16]. There is a vast range of methodologies that can be used to study biofilms *in vitro*, covering different aspects from biofilm structure and biomass, to biofilm composition and viability [17]. However, little is known about which techniques are more appropriate and have been mostly used to study phage efficacy and how the selected conditions can affect the outcomes. The aim of the present review was to study the influence of different biofilm and phage parameters on *in vitro* biofilm control by phages, focusing on the removal of pre-formed biofilms. To the best of our knowledge, this is the first systematic review gathering information on the use of phages to control bacterial biofilms *in vitro*. Consequently, we also aim at providing an overview of the strategies that have been employed over the last years to study the action of phages on biofilms *in vitro*, as well as recommendations for future work on this topic.

## Materials and methods

### Search strategy

Potential eligible articles were identified through a search conducted in three electronic databases: Web of Science, Embase, and Medline (PubMed). The search was restricted to articles written in English and published between 1 January 2000 and 19 July 2021, excluding review articles. The following terms were used to perform the electronic search: (((phages) OR (bacteriophages)) AND (biofilm) AND (*in vitro*)). This systematic review was guided by the PRISMA (Preferred Reporting Items for Systematic Reviews and Meta-Analyses) guidelines [18] (see Table S1 in Online Resource 1 for PRISMA checklist), and a review protocol was not prospectively prepared and registered.

### Selection criteria

The records obtained after conducting the search on each database were exported to EndNote for automatic exclusion of duplicates. Title and abstract screening were conducted manually to select the relevant articles, subsequently evaluated by full text screening. Only original articles published in peer-reviewed journals and containing data on the *in vitro* use of a single phage solution to control a pre-formed single-species biofilm, were included in this review. Data from studies with multispecies biofilms, the combination of more than one phage in a cocktail solution, and the inhibition of biofilm formation were excluded. Also, studies performed *in vivo* or *ex vivo*, with the use of prophages or phage-derived products (such as endolysins), and with the use of phages combined with other compounds or products (e.g. antibiotics) were not included in this review. These selection and exclusion criteria have been used to focus specifically on studying the individual activity of phages to control pre-formed biofilms *in vitro*, in order to better understand which phage parameters influence the biofilm reduction outcome.

Two authors (A.B. and L.M.) independently performed title, abstract, and full text screening, using the eligibility criteria described. Disagreements about the excluded articles were resolved by two different authors (D.P.P. and J.A.).

### Data extraction and analysis

Data collection from the articles was performed independently by two authors (A.B. and L.M.). From each study selected for inclusion in the systematic review, the following information was collected: biofilm growth conditions (bacterial species, *in vitro* biofilm formation surface, biofilm age, biofilm growth medium, with or without agitation), phage characteristics (family/morphology, genome size, burst size, latent period), treatment conditions (solution used for phage application, phage concentration, infection time) and treatment outcome (biofilm quantification method, biofilm reduction values) (see Table S2 in Online Resource 1 for the definition of each variable). These data were directly recovered from the text, converted to the same units or, in some cases, inferred from graphical analysis.

The statistical analysis was performed using R 4.2.2. [19]. Data extracted from the articles was condensed in a Microsoft Excel table and summarized through descriptive statistics, using bar charts, pie charts and box plots for quantitative variables and frequency tables for qualitative variables. Comparative analysis to assess the effect of the different variables on biofilm reduction was performed using Students t-test (for two independent samples) or its non-parametric equivalent Mann–Whitney U test when the assumption of normality was not met. For more than two independent samples, the method selected was one-way analysis of variance (ANOVA) (in case of normality of response variable) or its non-parametric equivalent Kruskal–Wallis Test. Multiple comparisons tests were conducted after the identification of statistically significant differences. The assumption of normality (quantitative variables) was examined by Shapiro–Wilk test. Population associated studies were conducted using Chi-Square Test of Independence for categorical variables and Spearman's Rank Correlation Coefficient to assess functional associations. The decision rule consisted of detecting statistically significant evidence for probability values (*p*-value) less than 0.05.

## Results

### Characteristics of included studies

**Fig. 1:**
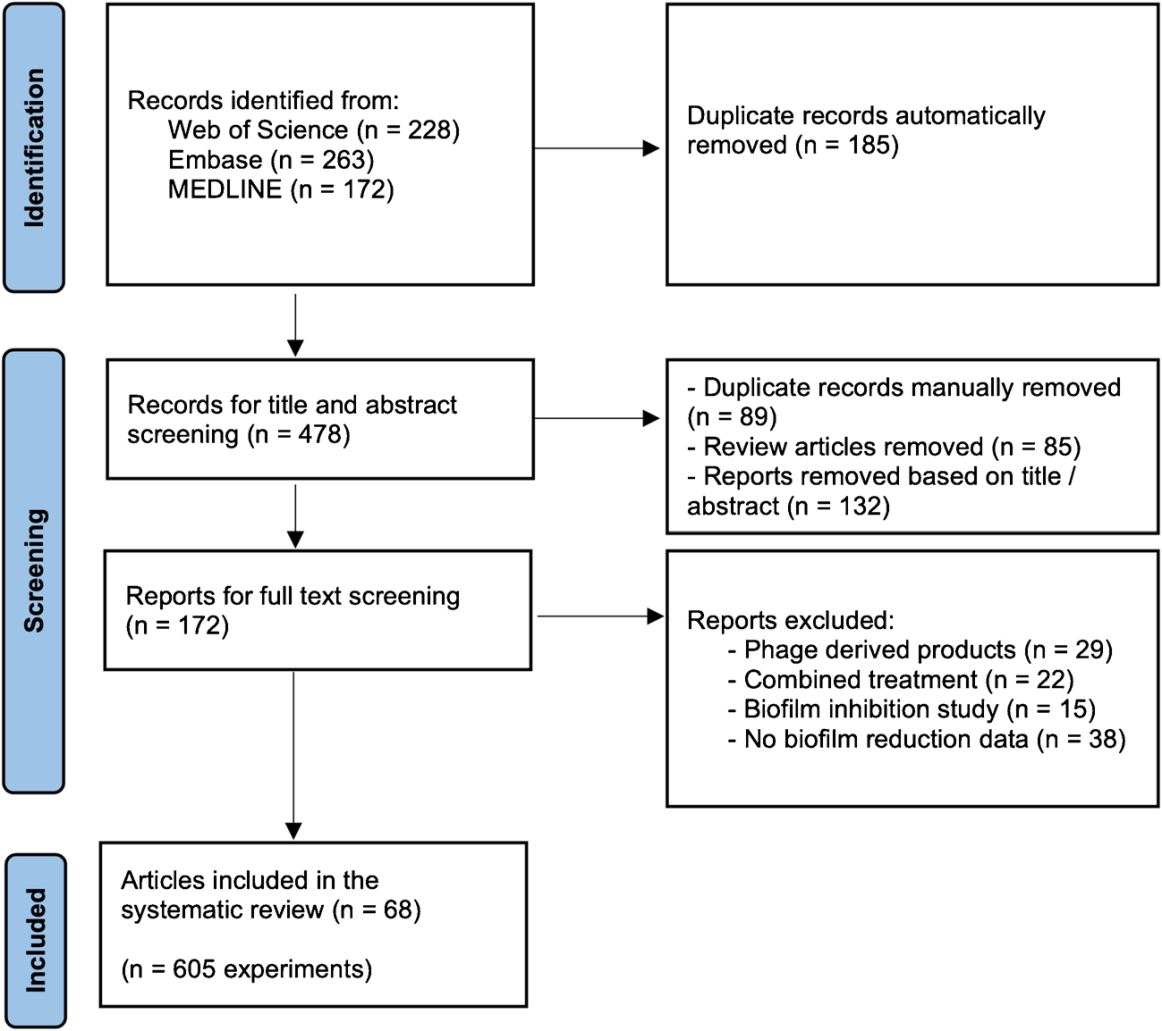
Flow-chart of literature screening process

The online search retrieved 663 records (Fig. 1). After automatic removal of duplicates using EndNote, 478 records remained for analysis. After manual curation it was possible to remove additional duplicates (*n* = 89) as well as review articles (*n* = 85). Based on title and abstract screening, 132 records were removed because they did not meet the inclusion criteria, leaving 172 for full text screening. Of those, 104 studies were excluded due to the use of phage-derived products (*n* = 29), use of phage cocktails or phages in combination with other antimicrobials (*n* = 22), use of phages to prevent biofilm formation (*n* = 15), and lack of biofilm reduction data (*n* = 38). A total of 68 studies met all the inclusion criteria and were included in the systematic review (see Table S3 in Online Resource 1 for full list of documents). From these selected articles, it was possible to retrieve data on 605 experiments of the use of single phages to control single-species bacterial biofilms *in vitro*.

### Biofilm growth conditions

The 605 experiments included data on biofilms formed by 19 different bacterial species, systematized in this review into the following groups: *Acinetobacter* spp. (3.0%), *Aeromonas* spp. (1.2%), *Aggregatibacter* spp. (2.0%), *Citrobacter* spp. (0.2%), *Clostridium* spp. (0.7%), *Enterococcus* spp. (9.1%), *Escherichia* spp. (10.4%), *Flavobacterium* spp. (4.8%), *Klebsiella* spp. (13.7%), *Proteus* spp. (9.1%), *Pseudomonas* spp. (21.5%), *Salmonella* spp. (9.4%), *Staphylococcus* spp. (13.4%), *Streptococcus* spp. (0.7%), and *Vibrio* spp. (1.0%) (Fig. S1).

The *in vitro* biofilm formation method was reported in 584 experiments (96.5%) (Fig. S2). In the majority of studies (82.5%), biofilms were formed on the surface of well plates. Other surfaces tested include catheter pieces (1.5%), glass beads (3.4%), glass coverslips (2.7%), nephrophane membranes (1.2%), polyvinyl chloride (PVC) coupons (0.3%) and stainless-steel coupons (7.7%). In 99.5% of all experiments a closed system was used; the experiments with the drip flow biofilm reactor correspond to the only case where a continuous flow system, or dynamic model, was used (0.5%).

The biofilm age used for phage treatment ranged from 12 to 672 h, with a median of 24 h. Different culture media have been used for biofilm formation (Fig. S3): brain heart infusion (BHI) (6.4%), cation-adjusted Mueller–Hinton broth (CAMHB) (5.0%), lysogeny broth (LB) (32.9%), minimum essential medium (MEM) (1.2%), Mueller–Hinton broth (MHB) (3.5%), Mueller–Hinton broth 2 (MHII) (0.2%), minimal medium (MM) (1.3%), nutrient broth (NB) (15.2%), Roswell Park Memorial Institute (RPMI) 1640 medium (0.2%), tryptic soy broth (TSB) (28.1%), TSB with fetal bovine serum and NaCl (SWF) (1.3%), and tryptone yeast extract salts (TYES) broth (4.8%). In some experiments, the culture media was supplemented with glucose. However, this distinction was not considered for the statical analysis. The use of agitation during biofilm formation and treatment with phages was observed for 24.3% of the experiments, although information on this variable was not present in 160 experiments (25.5%).

### Phage characteristics

Data regarding phage family was provided for 582 experiments (96.2%). Given the significant changes on phage taxonomy over the last years, with the creation of 7 new phage families between 2014 and 2019, it is possible to see different phage family designations in the articles during this transition period [20]. Therefore, the phages were categorized in this review according to their morphotypes into myovirus (33.7%), podovirus (32.5%), siphovirus (32.5%), and untailed icosahedral phages belonging to the *Microviridae* family (1.4%) (Fig. S4) [11].

Information about genome size, burst size and latent period was reported for 458 (75.7%), 434 (71.7%), and 435 (71.9%) experiments, respectively. The sizes of the genomes of the phages used varied from 5,386 to 286,783 bp, with a median of 44,194 bp. The median for the burst sizes was 70.0 PFU/infected cell and for the latent period it was 25.0 min.

### Treatment conditions

The solution used for phage application was reported in 600 experiments (99.2%) and categorized into buffer (28.5%), lysate (28.2%), rich media (40.7%), or others (2.7%) (including mammalian cell culture medium, minimum medium and saline solution) (Fig. S5). To compare the number of phages used for biofilm treatment, phage concentration was identified or calculated and converted to PFU/mL. It was possible to obtain data on phage concentration in 480 experiments (79.3%), in which it varied from 5 × 10^1^ PFU/mL to 5 × 10^12^ PFU/mL, with a median of 1 × 10^8^ PFU/mL. The infection time, reported in 603 experiments (99.7%), ranged from 1 to 168 h, with a median of 24 h.

### Treatment outcome

**Fig. 2:**
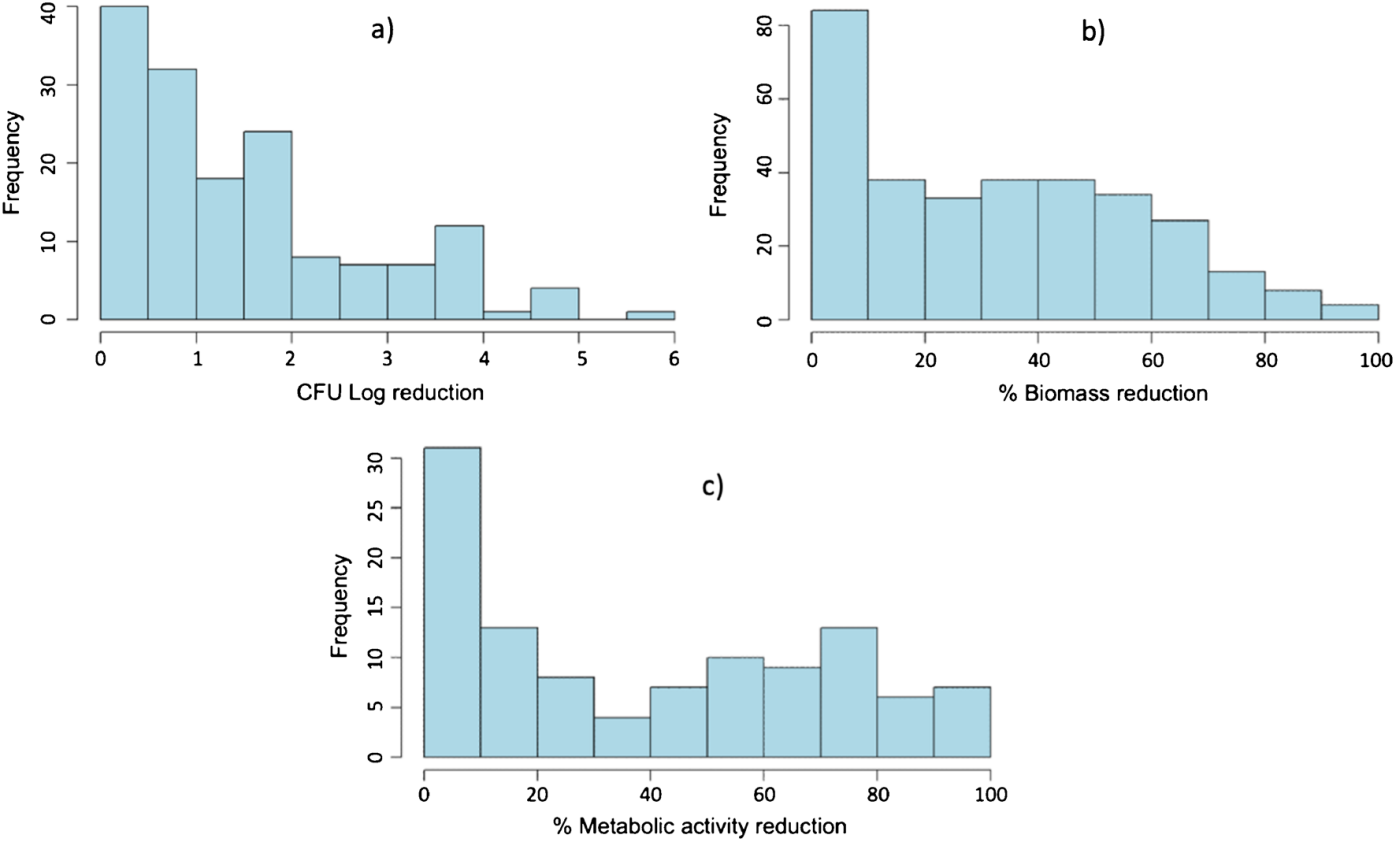
Distribution of the biofilm reduction values after phage treatment: CFU log reduction (**a**), percentage of biomass reduction (**b**), and percentage of metabolic activity reduction (**c**)

The methods used to evaluate the efficacy of phages for biofilm control included biomass quantification (52.6%; mainly by crystal violet assay), CFU counts (25.5%), metabolic activity (17.9%), and others (4.1%) (including molecular quantification, fluorescence, and laser interferometry) (Fig. S6). Percentage of biomass reduction, CFU Log reduction and percentage of metabolic activity reduction were obtained from the text or inferred from graphical analysis, comparing to the untreated controls. The value of 0 reduction was adopted for experiments without biofilm reduction. The distribution of biofilm reduction values for the 3 most common biofilm assessment methods identified is shown in Fig. 2. It is possible to observe a high variability of data, with most of the experiments resulting in a CFU log reduction between 0 and 2, and a biomass or metabolic activity reduction between 0 and 50%. It must be stressed that it is not possible to compare the three methods, as they are evaluating different outcomes. For instance, a high biomass reduction does not necessarily correspond to a high reduction in the viable cells, as phages can induce biofilm cells dispersion through the degradation of EPS components, independently of phage-induced cell lysis [21]. Therefore, it is important to consider the results from different biofilm assessment methods.

### Effect of the different variables on biofilm control by phages

**Fig. 3:**
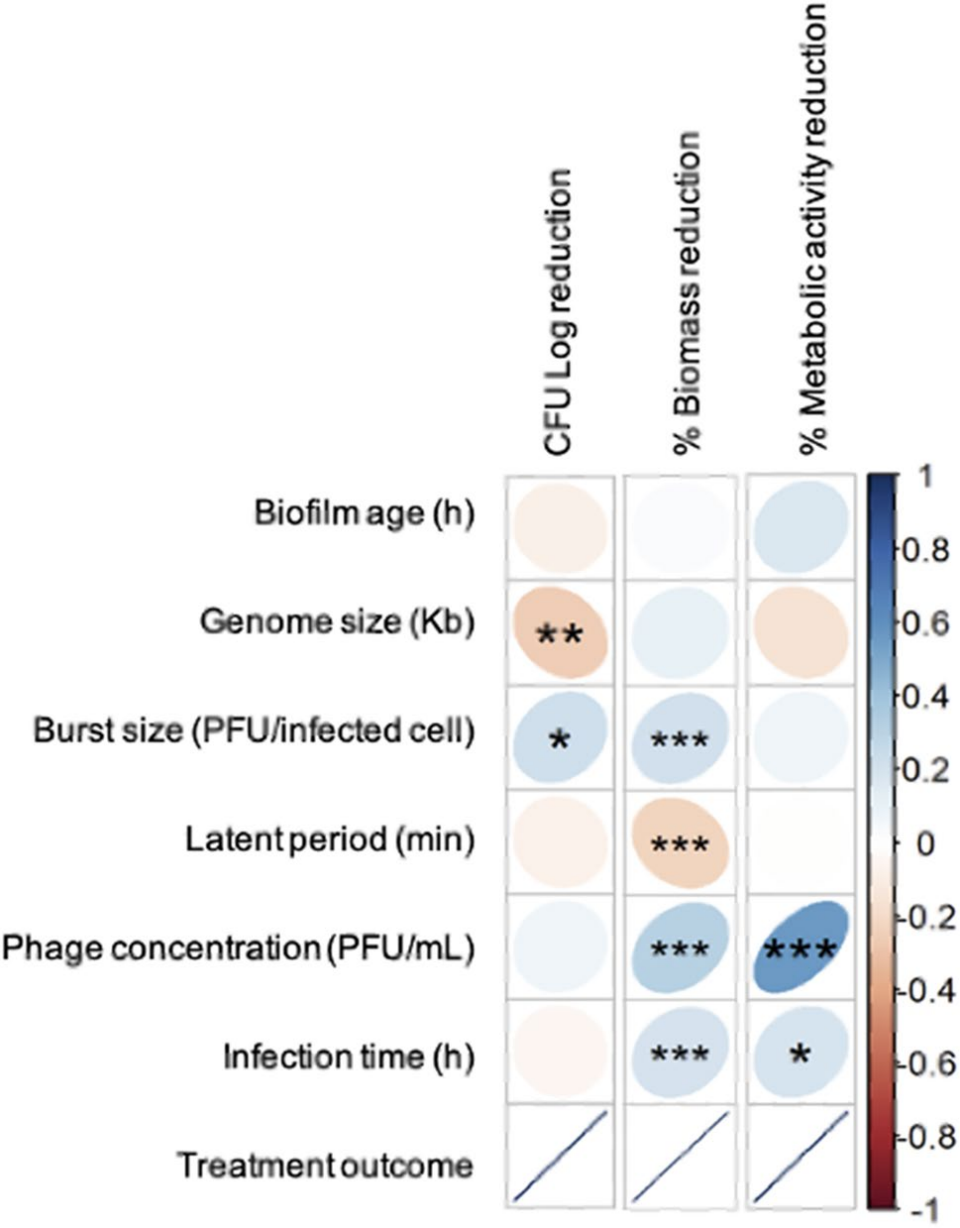
Correlation between the continuous variables (biofilm age, genome size, burst size, latent period, phage concentration, and infection time) and biofilm reduction as stated by the analysis of viable cell counts, biomass, or metabolic activity. Statistically significant correlations between each variable are marked with * for *p* < 0.05, ** *p* < 0.01, and *** for *p* < 0.001

Correlation analysis led to the identification of statistically significant relations between some continuous variables and the outcomes obtained by the different biofilm assessment methods (Fig. 3). Smaller phage genome sizes (r_S_ =—0.254, *p* < 0.01) and higher burst sizes (r_S_ = 0.222, *p* < 0.05) are associated with greater biofilm reduction measured by CFU counts. After the assessment of biofilm biomass, higher levels of biofilm reduction are associated with higher phage burst sizes (r_S_ = 0.214, *p* < 0.001), lower phage latent periods (r_S_ =—0.222, *p* < 0.001), higher phage concentrations (r_S_ = 0.318, *p* < 0.001), and higher infection times (r_S_ = 0.206, *p* < 0.001). When metabolic activity was used as a measure, the biofilm reduction is more pronounced for higher phage concentrations (r_S_ = 0.563, *p* < 0.001) and higher infection times (r_S_ = 0.197, *p* < 0.05). Therefore, smaller genome sizes lead to better treatment outcomes, but only for CFU reduction. For burst size and latent period, the correlations found are coherent among the different biofilm assessment methods, with a greater outcome being stimulated by higher burst sizes (statistically significant for CFU and biomass quantification) and shorter latent periods (statistically significant for biomass quantification) (Fig. 3).

Regarding the discrete variables, there were no differences between bacterial species (Fig. S7), *in vitro* biofilm formation surface (Fig. S8), and the use of agitation (Fig. S9) on biofilm reduction. Statistically significant differences were identified for biofilm growth medium (Fig. S10), phage morphology (Fig. S11), and solution for phage application (Fig. S12). Regarding the biofilm growth medium, although the use of BHI led to higher values of biomass reduction comparing to the other culture media (*p* < 0.001), the same tendency was not observed for CFU log reduction and percentage of metabolic activity reduction (Fig. S10). It was not possible to determine the phage morphology that leads to better treatment outcomes, since there was no consistency between the results of the different assessment methods, with podoviruses having the best performance in CFU log reduction (Fig. S11a) (*p* < 0.001) but the worst results in biomass reduction (Fig. S11b) (*p* < 0.001). Concerning the solution for phage application, the use of phage lysate was associated with higher CFU Log reduction (Fig. S12a) (*p* < 0.001), while the use of rich media was associated with higher percentage of biomass reduction (Fig. S12b) (*p* < 0.001) and higher percentage of metabolic activity reduction (Fig. S12c) (p < 0.01).

## Discussion

This systematic review is based on all studies published between January 2000 and July 2021 that deal with the use of single phages to control single-species bacterial biofilms *in vitro*. A total of 68 articles were eligible for inclusion, providing data on 605 experiments of biofilm control by phages.

Different bacterial species have been used for biofilm formation, and the most predominant were the ones belonging to *Pseudomonas* spp., *Klebsiella* spp., *Staphylococcus* spp., *Escherichia* spp., *Salmonella* spp., *Proteus* spp. and *Enterococcus* spp.. These bacteria are associated with life-threatening nosocomial infections, usually characterized by high levels of antibiotic resistance, and include some of the leading pathogens responsible for the global deaths attributable to antimicrobial resistance [22–24]. Besides the implications in healthcare, *Salmonella* spp. biofilms are an important concern in agricultural and food industries [8, 25], where the emergence of antibiotic-resistant strains also stimulates the interest in the use of phages as biocontrol agents [26].

The biofilms studied in the experiments analyzed in this review were mainly formed in the surface of microtiter plates (82.5%). This method allows a high throughput testing of multiple variables at the same time and can be adapted to simulate different biofilm-forming conditions, by changing different parameters including incubation time, temperature, and/or agitation [17]. Also, different biofilm assessment methods can be applied in microtiter plates, with good reproducibility, allowing the comparison of results from different labs [27]. However, since no guidelines to assess the efficacy of phages against biofilms formed in microtiter plates were developed so far, there is a high variability among the biofilm formation parameters across the different studies analyzed, which can influence the biofilm architecture and, consequently, the outcome of phage treatment. Although most of phage infection experiments were performed in biofilms grown for 24 h, the biofilm age varied from 12 to 672 h. The older biofilms were used to simulate the activity of phages on aquatic biofilms [28], and the younger biofilm were used to mimic a clinical infection [29]. It is important to consider the biofilm formation time and adjust it according to the *in vivo* condition under study, also taking into account that older biofilms tend to be more difficult to eradicate [30, 31]. It is also known that the flow conditions influence various aspects of the biofilm life cycle, including growth rate, rate of detachment and disaggregation [32]. For instance, shear stress promoted by agitation influences biofilm structure and metabolic activity, which consequently can impact the phage killing efficacy [33–35]. Moreover, agitation can positively influence the phage distribution through the biofilm. However, dispersion of bacteria might also limit phage-bacteria interactions. Therefore, the use of different shaking conditions for biofilm growth and treatment can influence the effect of phages on biofilm removal. In this review, this parameter varied across the articles, with most of the experiments (75.7%) being performed under static conditions, and the remaining varying from 77 to 150 rpm.

Regarding the culture medium used for biofilm formation, a high tendency towards the use of laboratory media was observed (mostly LB, TSB and NB). However, these culture media are very different from the environmental or body fluids where biofilms are formed *in vivo*. Media simulating the *in vivo* microenvironment are likely more adequate to study the activity of phages against bacterial biofilms *in vitro*. For example, it has been shown that *P. aeruginosa* shows similar phenotypes when grown in cystic fibrosis sputum or in a synthetic cystic fibrosis sputum medium [36, 37]. Additionally, an artificial chronic wound medium showed *P. aeruginosa* and *S. aureus* cooperation *in vitro*, as commonly found in chronic wounds, but this bacterial association is very difficult to observe in standard culture media [38]. Moreover, a medium-specific response to phage infection was observed when comparing the transcriptional profile of *P. aeruginosa* after phage infection in LB and in a mammalian cell culturing medium, indicating that the bacterial growth media has a high impact on phage-bacteria interactions [39]. Therefore, the use of microtiter plates for biofilm studies should evolve towards the use of media mimicking the host environments. This would enable a better understanding of phage performance against biofilms in real conditions.

The morphology of the phages used to control the pre-formed biofilms was well distributed within the class of tailed phages *Caudoviricetes*, without significant differences between myoviruses, podoviruses and siphoviruses. The use of an untailed icosahedral phage was only reported in a single study [40]. These results are in line with what has been observed for phage therapeutic applications, with a predominant use of virulent tailed phages from the *Caudoviricetes* class [41]. Besides phage morphology, also genome size, burst size and latent period are important traits that can influence phage-host interactions [42] and phage activity against biofilms. Although the relevance of including this information in studies about phage-biofilm interactions, there was a lack of these data in more than 25% of the experiments included in this review.

The solution used for phage application should also mimic the solutions that can be used to apply phages in real conditions, where a high degree of purification is needed to avoid adverse effects [43]. However, most of the experiments applied phages using rich media and there was also a high prevalence of using phage lysate, which is not in accordance with the solutions used in human phage therapy. Phage concentration and infection time varied across studies. However, in most cases, the biofilms were treated for 24 h with phages at a concentration of 1 × 10^8^ PFU/mL. This concentration is in accordance with the concentrations generally used to treat biofilm-related infections in human patients [44]. However, improved *in vitro* studies and clinical trials are still needed to assess the most appropriate treatment regimens, including dosing and duration [45].

For the assessment of phage activity against biofilms, the majority of the experiments were based on the quantification of biofilm biomass using the crystal violet assay, quantification of viable cells by CFU counts and quantification of metabolic activity. These methods rely on different biofilm parameters, which means that their use as single methods to assess biofilm control by phages can lead to different outcomes. Therefore, in order to obtain an in depth view of the antibiofilm activity of a phage, different biofilm assessment methods should be combined [46, 47]. In only 10 of the 68 articles included in this review more than one biofilm assessment method was used, most frequently the combination of crystal violet and CFU counts [48–57]. Those 10 articles also complemented the results with microscopic analysis of the biofilms, an important additional method that can be combined with quantitative software to understand the impact of phages on biofilm structure, especially for suspended biofilms where the use of the traditional crystal violet assay is not possible [58–60]. Despite the biofilm method selected, CFU quantification should always be performed in parallel, as it has been shown to be the most reliable method to assess the antimicrobial efficacy, when compared to biomass quantification by crystal violet assay and evaluation of metabolic activity using resazurin [27].

The comparative analysis suggested that the anti-biofilm effect of phages is more influenced by phage characteristics and treatment conditions than by biofilm growth conditions, as no statistically significant influence has been identified for bacterial species, biofilm formation surface, biofilm age, and the use of agitation. The use of higher phage concentrations and longer treatment periods were associated with greater biofilm reduction both for biomass and metabolic activity quantification. The absence of a positive association between CFU counts and infection time might be a consequence of the emergence of phage-insensitive mutants for longer phage treatments, as observed after exposing *P. aeruginosa* biofilms to phages for periods longer than 6 h [61]. Overall, the results suggest that phages with higher burst sizes, and shorter latent periods, may be more efficient for biofilm control when applied at high concentrations. These results are in line with the mode of action of phages, as phages with shorter latent periods have a faster life cycle, which contributes to a faster replication to generate new phage particles. Also, higher burst sizes allow phages to quickly increase in concentration and eliminate bacteria in a shorter period, which can contribute to a lower risk of selection of phage resistant bacteria [62].

Given the high variability of data presentation among the analyzed publications, data collection and comparison were very difficult and in some occasions there was the need to calculate parameters using values obtained by extrapolations of data from graphs or conversion of units, which can lead to misinterpretations. Another limitation is related with the fact that in some articles, some of the parameters analyzed were missing, which weakens the statistical analysis of the results. The articles included in this review have been published until July 2021, as this was the date of the electronic search that preceded data uniformization and analysis, and no advances in the methodologies have been found in the articles published up to the writing of this work.

As far as we are aware, this is the first systematic review providing an overview and analysis of the use of phages to control biofilms *in vitro*. With the continuous and worldwide spread of antibiotic resistance, and the high potential of phages to control bacteria and biofilms in the environment and in healthcare, it is important to have a general idea of the results from *in vitro* studies that have been developed over the last years. Besides the knowledge about the way phages have been tested against biofilms *in vitro*, with opportunity for improvement, these results can help understanding the parameters that influence phage efficacy on biofilms.

To conclude, besides the great variability among the methods used to assess phage/biofilm interactions, phage biological properties are the variables with higher impact on biofilm control by phages. Nevertheless, given the high importance of phages as biofilm control agents and the relevance of systematic analysis of phage performance studies, there is a need for standardized and reproducible methods. Efforts on this topic have been made recently, however there are still no guidelines for phage-biofilm studies [63, 64]. Also, microplate-based studies should be improved to better mimic real conditions, so that the results can be extrapolated to predict phage efficacy *in vivo*. In the future, a similar systematic analysis could be used to better understand the efficacy of phages in preventing biofilm formation, or to study the efficacy of phages and antibiotics combination in biofilm prevention or control.

## Supplementary information

Below is the link to the electronic supplementary material.

Supplementary file1 (DOCX 560 KB)

## Data Availability

The datasets generated during and/or analysed during the current study are available from the corresponding author on reasonable request.
