# Supplementary material for: A systematic review of the use of bacteriophages for in vitro biofilm control
Source: Eur J Clin Microbiol Infect Dis. 2023 Jul 5;42(8):919–28. doi: 10.1007/s10096-023-04638-1 (PMC10345070; doi:10.1007/s10096-023-04638-1)
Supplement: Supplementary file 1 — Supplementary file1 (DOCX 560 KB) [file 10096_2023_4638_MOESM1_ESM.docx]

**A systematic review of the use of bacteriophages for *in vitro* biofilm control**

European Journal of Clinical Microbiology & Infectious Diseases

Luciana Meneses, Ana Catarina Brandão, Tom Coenye, Ana Cristina Braga, Diana Priscila Pires, Joana Azeredo

Corresponding author: Joana Azeredo, Centre of Biological Engineering, University of Minho (jazeredo@deb.uminho.pt)

**Supplementary Information**

**Table S1.** PRISMA checklist


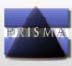
**PRISMA 2020 Checklist**

| **Section and Topic** | **Item #** | **Checklist item** | **Location where item is reported** |
| --- | --- | --- | --- |
| **TITLE** | | |  |
| Title | 1 | Identify the report as a systematic review. | Title |
| **ABSTRACT** | | |  |
| Abstract | 2 | See the PRISMA 2020 for Abstracts checklist. | Abstract |
| **INTRODUCTION** | | |  |
| Rationale | 3 | Describe the rationale for the review in the context of existing knowledge. | Paragraphs 1-3 |
| Objectives | 4 | Provide an explicit statement of the objective(s) or question(s) the review addresses. | Paragraph 4 |
| **METHODS** | | |  |
| Eligibility criteria | 5 | Specify the inclusion and exclusion criteria for the review and how studies were grouped for the syntheses. | Paragraph 1 |
| Information sources | 6 | Specify all databases, registers, websites, organisations, reference lists and other sources searched or consulted to identify studies. Specify the date when each source was last searched or consulted. | Paragraph 1 |
| Search strategy | 7 | Present the full search strategies for all databases, registers and websites, including any filters and limits used. | Paragraph 1 |
| Selection process | 8 | Specify the methods used to decide whether a study met the inclusion criteria of the review, including how many reviewers screened each record and each report retrieved, whether they worked independently, and if applicable, details of automation tools used in the process. | Paragraphs 2-3 |
| Data collection process | 9 | Specify the methods used to collect data from reports, including how many reviewers collected data from each report, whether they worked independently, any processes for obtaining or confirming data from study investigators, and if applicable, details of automation tools used in the process. | Paragraph 4 |
| Data items | 10a | List and define all outcomes for which data were sought. Specify whether all results that were compatible with each outcome domain in each study were sought (e.g. for all measures, time points, analyses), and if not, the methods used to decide which results to collect. | Paragraph 4 |
|  | 10b | List and define all other variables for which data were sought (e.g. participant and intervention characteristics, funding sources). Describe any assumptions made about any missing or unclear information. | Paragraph 4, Table S2 |
| Study risk of bias assessment | 11 | Specify the methods used to assess risk of bias in the included studies, including details of the tool(s) used, how many reviewers assessed each study and whether they worked independently, and if applicable, details of automation tools used in the process. | N/A |
| Effect measures | 12 | Specify for each outcome the effect measure(s) (e.g. risk ratio, mean difference) used in the synthesis or presentation of results. | N/A |
| Synthesis methods | 13a | Describe the processes used to decide which studies were eligible for each synthesis (e.g. tabulating the study intervention characteristics and comparing against the planned groups for each synthesis (item #5)). | N/A |
|  | 13b | Describe any methods required to prepare the data for presentation or synthesis, such as handling of missing summary statistics, or data conversions. | Paragraph 4 |
|  | 13c | Describe any methods used to tabulate or visually display results of individual studies and syntheses. | Paragraph 5 |
|  | 13d | Describe any methods used to synthesize results and provide a rationale for the choice(s). If meta-analysis was performed, describe the model(s), method(s) to identify the presence and extent of statistical heterogeneity, and software package(s) used. | Paragraph 5 |
|  | 13e | Describe any methods used to explore possible causes of heterogeneity among study results (e.g. subgroup analysis, meta-regression). | Paragraph 5 |
|  | 13f | Describe any sensitivity analyses conducted to assess robustness of the synthesized results. | Paragraph 5 |
| Reporting bias assessment | 14 | Describe any methods used to assess risk of bias due to missing results in a synthesis (arising from reporting biases). | N/A |
| Certainty assessment | 15 | Describe any methods used to assess certainty (or confidence) in the body of evidence for an outcome. | Paragraph 5 |
| **RESULTS** | | |  |
| Study selection | 16a | Describe the results of the search and selection process, from the number of records identified in the search to the number of studies included in the review, ideally using a flow diagram. | Paragraph 1, Fig. 1 |
|  | 16b | Cite studies that might appear to meet the inclusion criteria, but which were excluded, and explain why they were excluded. | N/A |
| Study characteristics | 17 | Cite each included study and present its characteristics. | Table S3 |
| Risk of bias in studies | 18 | Present assessments of risk of bias for each included study. | N/A |
| Results of individual studies | 19 | For all outcomes, present, for each study: (a) summary statistics for each group (where appropriate) and (b) an effect estimate and its precision (e.g. confidence/credible interval), ideally using structured tables or plots. | Paragraphs 2-10, |
| Results of syntheses | 20a | For each synthesis, briefly summarise the characteristics and risk of bias among contributing studies. | N/A |
|  | 20b | Present results of all statistical syntheses conducted. If meta-analysis was done, present for each the summary estimate and its precision (e.g. confidence/credible interval) and measures of statistical heterogeneity. If comparing groups, describe the direction of the effect. | Paragraphs 2-10 |
|  | 20c | Present results of all investigations of possible causes of heterogeneity among study results. | N/A |
|  | 20d | Present results of all sensitivity analyses conducted to assess the robustness of the synthesized results. | N/A |
| Reporting biases | 21 | Present assessments of risk of bias due to missing results (arising from reporting biases) for each synthesis assessed. | N/A |
| Certainty of evidence | 22 | Present assessments of certainty (or confidence) in the body of evidence for each outcome assessed. | N/A |
| **DISCUSSION** | | |  |
| Discussion | 23a | Provide a general interpretation of the results in the context of other evidence. | Paragraphs 2-8 |
|  | 23b | Discuss any limitations of the evidence included in the review. | Paragraph 9 |
|  | 23c | Discuss any limitations of the review processes used. | Paragraph 9 |
|  | 23d | Discuss implications of the results for practice, policy, and future research. | Paragraphs 10,11 |
| **OTHER INFORMATION** | | |  |
| Registration and protocol | 24a | Provide registration information for the review, including register name and registration number, or state that the review was not registered. | Materials – Paragraph 1 |
|  | 24b | Indicate where the review protocol can be accessed, or state that a protocol was not prepared. | Materials – Paragraph 1 |
|  | 24c | Describe and explain any amendments to information provided at registration or in the protocol. | N/A |
| Support | 25 | Describe sources of financial or non-financial support for the review, and the role of the funders or sponsors in the review. | Acknowledgements |
| Competing interests | 26 | Declare any competing interests of review authors. | Declaration of competing interest |
| Availability of data, code and other materials | 27 | Report which of the following are publicly available and where they can be found: template data collection forms; data extracted from included studies; data used for all analyses; analytic code; any other materials used in the review. | Data availability |

**Table S2.** Glossary

| **Biofilm age** | Period necessary to develop biofilm structures. The higher the time allowed for biofilm formation, the older the biofilm is. |
| --- | --- |
| ***In vitro* biofilm formation surface** | The surface/material used to form a biofilm *in vitro*, with specific characteristics including area, roughness, and shape. |
| **Burst size** | Average number of phage particles that are produced per one infected bacterium at the end of each life cycle. |
| **Genome size** | Size of a phage genome (DNA or RNA) in base pairs. |
| **Latent period** | The time that a phage takes to infect and reproduce in the bacterial cell, from the moment of injection of phage genome into the host cell until the bacterial lysis with release of new progeny phages. |
| **Phage family** | Phage taxonomical classification according to the International Committee on Taxonomy of Viruses (ICTV). |
| **Infection time** | Total time between the application of the phage to the biofilm and the end of the treatment, with evaluation of phage efficacy. |
| **Biofilm reduction** | Term referred to the treatment outcome, usually evaluated by comparing the characteristics of the biofilm after the phage challenge with a control that is treated only with the treatment solution without phages. |

**Table S3.** List of articles included in the systematic review

| **Reference** |
| --- |
| [S1] Duarte AC, Fernández L, de Maesschalck V, Gutiérrez D, Campelo AB, Briers Y, *et al*. Synergistic action of phage phiIPLA-RODI and lytic protein CHAPSH3b: a combination strategy to target *Staphylococcus aureus* biofilms. NPJ Biofilms Microbiomes 2021;7. https://doi.org/10.1038/s41522-021-00208-5.  [S2] Cornelissen A, Ceyssens PJ, T’Syen J, van Praet H, Noben JP, Shaburova O v., *et al*. The T7-related *Pseudomonas putida* phage φ15 displays virion-associated biofilm degradation properties. PLoS One 2011;6. https://doi.org/10.1371/JOURNAL.PONE.0018597.  [S3] Cornelissen A, Ceyssens PJ, Krylov VN, Noben JP, Volckaert G, Lavigne R. Identification of EPS-degrading activity within the tail spikes of the novel *Pseudomonas putida* phage AF. Virology 2012;434. https://doi.org/10.1016/j.virol.2012.09.030.  [S4] Maszewska A, Zygmunt M, Grzejdziak I, Różalski A. Use of polyvalent bacteriophages to combat biofilm of *Proteus mirabilis* causing catheter-associated urinary tract infections. J Appl Microbiol 2018;125. https://doi.org/10.1111/jam.14026.  [S5] Oliveira A, Sousa JC, Silva AC, Melo LDR, Sillankorva S. Chestnut honey and bacteriophage application to control *Pseudomonas aeruginosa* and *Escherichia coli* biofilms: Evaluation in an *ex vivo* wound model. Front Microbiol 2018;9. https://doi.org/10.3389/fmicb.2018.01725.  [S6] Papadopoulou A, Dalsgaard I, Wiklund T. Inhibition Activity of Compounds and Bacteriophages against *Flavobacterium psychrophilum* Biofilms In Vitro. J Aquat Anim Health 2019;31. https://doi.org/10.1002/aah.10069.  [S7] Karaca B, Akcelik N, Akcelik M. Effects of P22 bacteriophage on *Salmonella enterica* subsp. enyerica serovar Typhimurium DMC4 strain biofilm formation and eradication. Arch Biol Sci 2015;67. https://doi.org/10.2298/ABS141120114K.  [S8] Pallavi B, Puneeth TG, Shekar M, Girisha SK. Isolation, characterization and genomic analysis of vB-AhyM-AP1, a lytic bacteriophage infecting *Aeromonas hydrophila*. J Appl Microbiol 2021;131. https://doi.org/10.1111/jam.14997.  [S9] Tao C, Yi Z, Zhang Y, Wang Y, Zhu H, Afayibo DJA, *et al*. Characterization of a Broad-Host-Range Lytic Phage SHWT1 Against Multidrug-Resistant Salmonella and Evaluation of Its Therapeutic Efficacy *in vitro* and *in vivo*. Front Vet Sci 2021;8. https://doi.org/10.3389/fvets.2021.683853.  [S10] Milho C, Silva MD, Melo L, Santos S, Azeredo J, Sillankorva S. Control of *Salmonella Enteritidis* on food contact surfaces with bacteriophage PVP-SE2. Biofouling 2018;34. https://doi.org/10.1080/08927014.2018.1501475.  [S11] Lemon DJ, Kay MK, Titus JK, Ford AA, Chen W, Hamlin NJ, *et al*. Construction of a genetically modified T7Select phage system to express the antimicrobial peptide 1018. Journal of Microbiology 2019;57. https://doi.org/10.1007/s12275-019-8686-6.  [S12] Kumaran D, Taha M, Yi QL, Ramirez-Arcos S, Diallo JS, Carli A, *et al*. Does treatment order matter? Investigating the ability of bacteriophage to augment antibiotic activity against *Staphylococcus aureus* biofilms. Front Microbiol 2018;9. https://doi.org/10.3389/fmicb.2018.00127.  [S13] Maura D, Morello E, du Merle L, Bomme P, le Bouguénec C, Debarbieux L. Intestinal colonization by enteroaggregative *Escherichia coli* supports long-term bacteriophage replication in mice. Environ Microbiol 2012;14. https://doi.org/10.1111/j.1462-2920.2011.02644.x.  [S14] Sasikala D, Srinivasan P. Characterization of potential lytic bacteriophage against *Vibrio alginolyticus* and its therapeutic implications on biofilm dispersal. Microb Pathog 2016;101. https://doi.org/10.1016/j.micpath.2016.10.017.  [S15] González-Villalobos E, Ribas-Aparicio RM, Montealegre GER, Belmont-Monroy L, Ortega-García Y, Aparicio-Ozores G, *et al*. Isolation and characterization of novel bacteriophages as a potential therapeutic option for *Escherichia coli* urinary tract infections. Appl Microbiol Biotechnol 2021;105. https://doi.org/10.1007/s00253-021-11432-6.  [S16] Townsend EM, Moat J, Jameson E. CAUTI’s next top model – Model dependent *Klebsiella* biofilm inhibition by bacteriophages and antimicrobials. Biofilm 2020;2. https://doi.org/10.1016/j.bioflm.2020.100038.  [S17] Yüksel FN, Buzrul S, Akçelik M, Akçelik N. Inhibition and eradication of *Salmonella Typhimurium* biofilm using P22 bacteriophage, EDTA and nisin. Biofouling 2018;34. https://doi.org/10.1080/08927014.2018.1538412.  [S18] Scarascia G, Yap SA, Kaksonen AH, Hong PY. Bacteriophage infectivity against *Pseudomonas aeruginosa* in saline conditions. Front Microbiol 2018;9. https://doi.org/10.3389/fmicb.2018.00875.  [S19] Topka-Bielecka G, Nejman-Faleńczyk B, Bloch S, Dydecka A, Necel A, Węgrzyn A, *et al*. Phage–Bacteria Interactions in Potential Applications of Bacteriophage vB_EfaS-271 against *Enterococcus faecalis*. Viruses 2021, Vol 13, Page 318 2021;13:318. https://doi.org/10.3390/V13020318.  [S20] Ben-Zaken H, Kraitman R, Coppenhagen-Glazer S, Khalifa L, Alkalay-Oren S, Gelman D, *et al*. Isolation and characterization of *Streptococcus mutans* phage as a possible treatment agent for caries. Viruses 2021;13. https://doi.org/10.3390/v13050825.  [S21] Oh HK, Hwang YJ, Hong HW, Myung H. Comparison of *Enterococcus faecalis* biofilm removal efficiency among bacteriophage pbef129, its endolysin, and cefotaxime. Viruses 2021;13. https://doi.org/10.3390/v13030426.  [S22] Duc HM, Son HM, Ngan PH, Sato J, Masuda Y, Honjoh K ichi, *et al*. Isolation and application of bacteriophages alone or in combination with nisin against planktonic and biofilm cells of *Staphylococcus aureus*. Appl Microbiol Biotechnol 2020;104. https://doi.org/10.1007/s00253-020-10581-4.  [S23] Dickey J, Perrot V. Adjunct phage treatment enhances the effectiveness of low antibiotic concentration against *Staphylococcus aureus* biofilms *in vitro*. PLoS One 2019;14. https://doi.org/10.1371/journal.pone.0209390.  [S24] Mendes JJ, Leandro C, Mottola C, Barbosa R, Silva FA, Oliveira M, *et al*. *In vitro* design of a novel lytic bacteriophage cocktail with therapeutic potential against organisms causing diabetic foot infections. J Med Microbiol 2014;63. https://doi.org/10.1099/jmm.0.071753-0.  [S25] Jeon J, Yong D. Two novel bacteriophages improve survival in *Galleria mellonella* infection and mouse acute pneumonia models infected with extensively drug-resistant *Pseudomonas aeruginosa*. Appl Environ Microbiol 2019;85. https://doi.org/10.1128/AEM.02900-18.  [S26] Liu J, Gao S, Dong Y, Lu C, Liu Y. Isolation and characterization of bacteriophages against virulent *Aeromonas hydrophila*. BMC Microbiol 2020;20. https://doi.org/10.1186/s12866-020-01811-w.  [S27] Nale JY, Chutia M, Carr P, Hickenbotham PT, Clokie MRJ. “Get in early”; Biofilm and wax moth (*Galleria mellonella*) models reveal new insights into the therapeutic potential of *Clostridium difficile* bacteriophages. Front Microbiol 2016;7. https://doi.org/10.3389/fmicb.2016.01383.  [S28] Danis-Wlodarczyk K, Vandenheuvel D, Jang H bin, Briers Y, Olszak T, Arabski M, *et al*. A proposed integrated approach for the preclinical evaluation of phage therapy in *Pseudomonas* infections. Sci Rep 2016;6. https://doi.org/10.1038/srep28115.  [S29] Dakheel KH, Rahim RA, Neela VK, Al-Obaidi JR, Hun TG, Isa MNM, *et al*. Genomic analyses of two novel biofilm-degrading methicillin-resistant *Staphylococcus aureus* phages. BMC Microbiol 2019;19. https://doi.org/10.1186/s12866-019-1484-9.  [S30] Sundell K, Landor L, Castillo D, Middelboe M, Wiklund T. Bacteriophages as Biocontrol Agents for *Flavobacterium psychrophilum* Biofilms and Rainbow Trout Infections. PHAGE: Therapy, Applications, and Research 2020;1. https://doi.org/10.1089/phage.2020.0021.  [S31] Jia K, Yang N, Zhang X, Cai R, Zhang Y, Tian J, *et al*. Genomic, Morphological and Functional Characterization of Virulent Bacteriophage IME-JL8 Targeting *Citrobacter freundii*. Front Microbiol 2020;11. https://doi.org/10.3389/fmicb.2020.585261.  [S32] Carson L, Gorman SP, Gilmore BF. The use of lytic bacteriophages in the prevention and eradication of biofilms of *Proteus mirabilis* and *Escherichia coli*. FEMS Immunol Med Microbiol 2010;59. https://doi.org/10.1111/j.1574-695X.2010.00696.x.  [S33] Melo LDR, Pinto G, Oliveira F, Vilas-Boas D, Almeida C, Sillankorva S, *et al*. The protective effect of *Staphylococcus epidermidis* biofilm matrix against phage predation. Viruses 2020;12. https://doi.org/10.3390/v12101076.  [S34] Melo LDR, Ferreira R, Costa AR, Oliveira H, Azeredo J. Efficacy and safety assessment of two enterococci phages in an *in vitro* biofilm wound model. Sci Rep 2019;9. https://doi.org/10.1038/s41598-019-43115-8.  [S35] Cobb LH, Park JY, Swanson EA, Beard MC, McCabe EM, Rourke AS, *et al*. CRISPR-Cas9 modified bacteriophage for treatment of *Staphylococcus aureus* induced osteomyelitis and soft tissue infection. PLoS One 2019;14. https://doi.org/10.1371/journal.pone.0220421.  [S36] Khalifa L, Gelman D, Shlezinger M, Dessal AL, Coppenhagen-Glazer S, Beyth N, *et al*. Defeating antibiotic- and phage-resistant *Enterococcus faecalis* using a phage Cocktail *in vitro* and in a clot model. Front Microbiol 2018;9. https://doi.org/10.3389/fmicb.2018.00326.  [S37] Khalifa L, Brosh Y, Gelman D, Coppenhagen-Glazer S, Beyth S, Poradosu-Cohen R, *et al*. Targeting *Enterococcus faecalis* biofilms with phage therapy. Appl Environ Microbiol 2015;81. https://doi.org/10.1128/AEM.00096-15.  [S38] Jiang L, Tan J, Hao Y, Wang Q, Yan X, Wang D, *et al*. Isolation and Characterization of a Novel Myophage Abp9 Against Pandrug Resistant *Acinetobacater baumannii*. Front Microbiol 2020;11. https://doi.org/10.3389/fmicb.2020.506068.  [S39] Al-Zubidi M, Widziolek M, Court EK, Gains AF, Smith RE, Ansbro K, *et al*. Identification of novel bacteriophages with therapeutic potential that target *Enterococcus faecalis*. Infect Immun 2019;87. https://doi.org/10.1128/IAI.00512-19.  [S40] Castillo-Ruiz M, Vinés ED, Montt C, Fernández J, Delgado JM, Hormazábal JC, *et al*. Isolation of a novel *Aggregatibacter actinomycetemcomitans* serotype b bacteriophage capable of lysing bacteria within a biofilm. Appl Environ Microbiol 2011;77. https://doi.org/10.1128/AEM.02115-10.  [S41] El-Telbany M, El-Didamony G, Askora A, Ariny E, Abdallah D, Connerton IF, *et al*. Bacteriophages to control multi-drug resistant *Enterococcus faecalis* infection of dental root canals. Microorganisms 2021;9. https://doi.org/10.3390/microorganisms9030517.  [S42] Li M, Shi D, Li Y, Xiao Y, Chen M, Chen L, *et al*. Recombination of T4-like Phages and Its Activity against Pathogenic *Escherichia coli* in Planktonic and Biofilm Forms. Virol Sin 2020;35. https://doi.org/10.1007/s12250-020-00233-2.  [S43] D’Andrea MM, Frezza D, Romano E, Marmo P, Henrici De Angelis L, Perini N, *et al*. The lytic bacteriophage vB_EfaH_EF1TV, a new member of the *Herelleviridae* family, disrupts biofilm produced by *Enterococcus faecalis* clinical strains. J Glob Antimicrob Resist 2020;21. https://doi.org/10.1016/j.jgar.2019.10.019.  [S44] Cerca N, Oliveira R, Azeredo J. Susceptibility of *Staphylococcus epidermidis* planktonic cells and biofilms to the lytic action of staphylococcus bacteriophage K. Lett Appl Microbiol 2007;45. https://doi.org/10.1111/j.1472-765X.2007.02190.x.  [S45] Taha OA, Connerton PL, Connerton IF, El-Shibiny A. Bacteriophage ZCKP1: A potential treatment for *Klebsiella pneumoniae* isolated from diabetic foot patients. Front Microbiol 2018;9. https://doi.org/10.3389/fmicb.2018.02127.  [S46] Ni P, Wang L, Deng B, Jiu S, Ma C, Zhang C, *et al*. Combined application of bacteriophages and carvacrol in the control of *Pseudomonas syringae* pv. *Actinidiae* planktonic and biofilm forms. Microorganisms 2020;8. https://doi.org/10.3390/microorganisms8060837.  [S47] Chang RYK, Das T, Manos J, Kutter E, Morales S, Chan HK. Bacteriophage PEV20 and Ciprofloxacin Combination Treatment Enhances Removal of *Pseudomonas aeruginosa* Biofilm Isolated from Cystic Fibrosis and Wound Patients. AAPS Journal 2019;21. https://doi.org/10.1208/s12248-019-0315-0.  [S48] Ahiwale S, Tamboli N, Thorat K, Kulkarni R, Ackermann H, Kapadnis B. *In vitro* management of hospital *Pseudomonas aeruginosa* biofilm using indigenous T7-like lytic phage. Curr Microbiol 2011;62. https://doi.org/10.1007/s00284-010-9710-6.  [S49] Bhardwaj SB, Mehta M, Sood S, Sharma J. Isolation of a novel phage and targeting biofilms of drug-resistant oral *enterococci*. J Glob Infect Dis 2020;12. https://doi.org/10.4103/jgid.jgid_110_19.  [S50] Kim SG, Giri SS, Yun S, Kim HJ, Kim SW, Kang JW, *et al*. Synergistic phage–surfactant combination clears IgE-promoted *Staphylococcus aureus* aggregation *in vitro* and enhances the effect *in vivo*. Int J Antimicrob Agents 2020;56. https://doi.org/10.1016/j.ijantimicag.2020.105997.  [S51] Latz S, Krüttgen A, Häfner H, Buhl EM, Ritter K, Horz HP. Differential effect of newly isolated phages belonging to PB1-like, phiKZ-like and LUZ24-like viruses against multi-drug resistant *Pseudomonas aeruginosa* under varying growth conditions. Viruses 2017;9. https://doi.org/10.3390/v9110315.  [S52] Liu S, Zhao Y, Hayes A, Hon K, Zhang G, Bennett C, *et al*. Overcoming bacteriophage insensitivity in *Staphylococcus aureus* using clindamycin and azithromycinat subinhibitory concentrations. Allergy: European Journal of Allergy and Clinical Immunology 2021;76. https://doi.org/10.1111/all.14883.  [S53] Manoharadas S, Altaf M, Alrefaei AF, Hussain SA, Devasia RM, Badjah Hadj AYM, *et al*. Microscopic analysis of the inhibition of staphylococcal biofilm formation by *Escherichia coli* and the disruption of preformed staphylococcal biofilm by bacteriophage. Microsc Res Tech 2021;84. https://doi.org/10.1002/jemt.23707.  [S54] Singla S, Harjai K, Katare OP, Chhibber S. Encapsulation of bacteriophage in liposome accentuates its entry in to macrophage and shields it from neutralizing antibodies. PLoS One 2016;11. https://doi.org/10.1371/journal.pone.0153777.  [S55] Olszak T, Danis-Wlodarczyk K, Arabski M, Gula G, MacIejewska B, Wasik S, *et al*. *Pseudomonas aeruginosa* PA5oct jumbo phage impacts planktonic and biofilm population and reduces its host virulence. Viruses 2019;11. https://doi.org/10.3390/v11121089.  [S56] Tkhilaishvili T, Wang L, Tavanti A, Trampuz A, di Luca M. Antibacterial Efficacy of Two Commercially Available Bacteriophage Formulations, Staphylococcal Bacteriophage and PYO Bacteriophage, Against Methicillin-Resistant *Staphylococcus aureus*: Prevention and Eradication of Biofilm Formation and Control of a Systemic Infection of *Galleria mellonella* Larvae. Front Microbiol 2020;11. https://doi.org/10.3389/fmicb.2020.00110.  [S57] Tkhilaishvili T, di Luca M, Abbandonato G, Maiolo EM, Klatt AB, Reuter M, *et al*. Real-time assessment of bacteriophage T3-derived antimicrobial activity against planktonic and biofilm-embedded *Escherichia coli* by isothermal microcalorimetry. Res Microbiol 2018;169. https://doi.org/10.1016/j.resmic.2018.05.010.  [S58] Magin V, Garrec N, Andrés Y. Selection of bacteriophages to control *in vitro* 24 h old biofilm of *Pseudomonas aeruginosa* isolated from drinking and thermal water. Viruses 2019;11. https://doi.org/10.3390/v11080749.  [S59] Verma V, Harjai K, Chhibber S. Restricting ciprofloxacin-induced resistant variant formation in biofilm of *Klebsiella pneumoniae* B5055 by complementary bacteriophage treatment. Journal of Antimicrobial Chemotherapy 2009;64. https://doi.org/10.1093/jac/dkp360.  [S60] Chaudhry WN, Concepcion-Acevedo J, Park T, Andleeb S, Bull JJ, Levin BR. Synergy and order effects of antibiotics and phages in killing *Pseudomonas aeruginosa* biofilms. PLoS One 2017;12. https://doi.org/10.1371/journal.pone.0168615.  [S61] Wang L, Tkhilaishvili T, Bernal Andres B, Trampuz A, Gonzalez Moreno M. Bacteriophage–antibiotic combinations against ciprofloxacin/ceftriaxone-resistant *Escherichia coli* *in vitro* and in an experimental *Galleria mellonella* model. Int J Antimicrob Agents 2020;56. https://doi.org/10.1016/j.ijantimicag.2020.106200.  [S62] Yan W, Banerjee P, Liu Y, Mi Z, Bai C, Hu H, *et al*. Development of thermosensitive hydrogel wound dressing containing *Acinetobacter baumannii* phage against wound infections. Int J Pharm 2021;602. https://doi.org/10.1016/j.ijpharm.2021.120508.  [S63] Wintachai P, Naknaen A, Pomwised R, Voravuthikunchai SP, Smith DR. Isolation and characterization of *Siphoviridae* phage infecting extensively drug-resistant *Acinetobacter baumannii* and evaluation of therapeutic efficacy *in vitro* and *in vivo*. J Med Microbiol 2019;68. https://doi.org/10.1099/jmm.0.001002.  [S64] Wintachai P, Naknaen A, Thammaphet J, Pomwised R, Phaonakrop N, Roytrakul S, *et al*. Characterization of extended-spectrum-β-lactamase producing *Klebsiella pneumoniae* phage KP1801 and evaluation of therapeutic efficacy *in vitro* and *in vivo*. Sci Rep 2020;10. https://doi.org/10.1038/s41598-020-68702-y.  [S65] Chen Y, Li X, Wang S, Guan L, Li X, Hu D, *et al*. A novel tail-associated o91-specific polysaccharide depolymerase from a podophage reveals lytic efficacy of Shiga Toxin-producing *Escherichia coli*. Appl Environ Microbiol 2020;86. https://doi.org/10.1128/AEM.00145-20.  [S66] Liu Y, Mi Z, Niu W, An X, Yuan X, Liu H, *et al*. Potential of a lytic bacteriophage to disrupt *Acinetobacter baumannii* biofilms *in vitro*. Future Microbiol 2016;11. https://doi.org/10.2217/fmb-2016-0104.  [S67] Guo Y, Chen P, Lin Z, Wang T. Characterization of two *Pseudomonas aeruginosa* viruses vB_PaeM_SCUT-S1 and vB_PaeM_SCUT-S2. Viruses 2019;11. https://doi.org/10.3390/v11040318.  [S68] Yazdi M, Bouzari M, Ghaemi EA. Isolation and Characterization of a Lytic Bacteriophage (vB_PmiS-TH) and Its Application in Combination with Ampicillin against Planktonic and Biofilm Forms of *Proteus mirabilis* Isolated from Urinary Tract Infection. J Mol Microbiol Biotechnol 2018;28:37–46. https://doi.org/10.1159/000487137. |


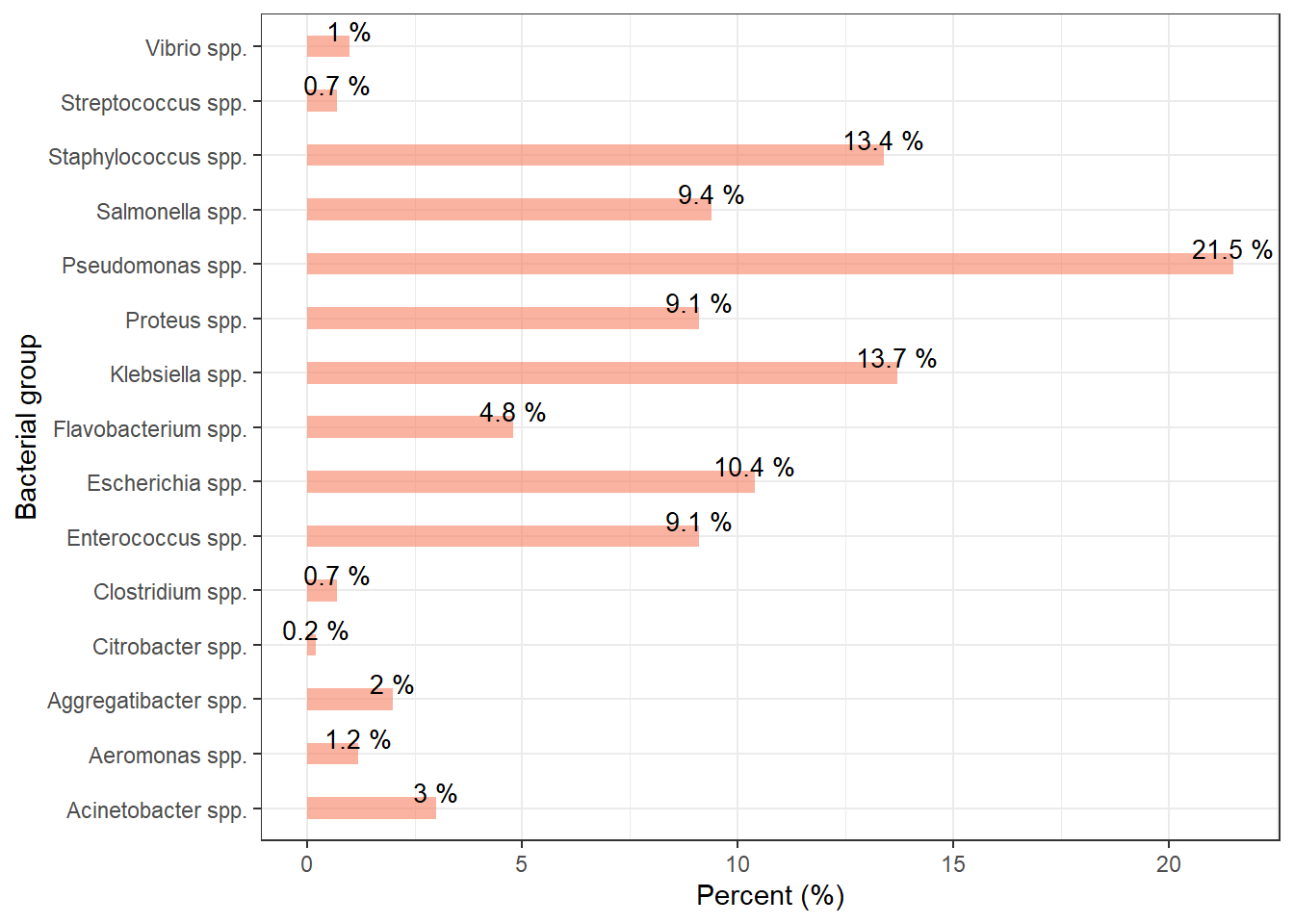

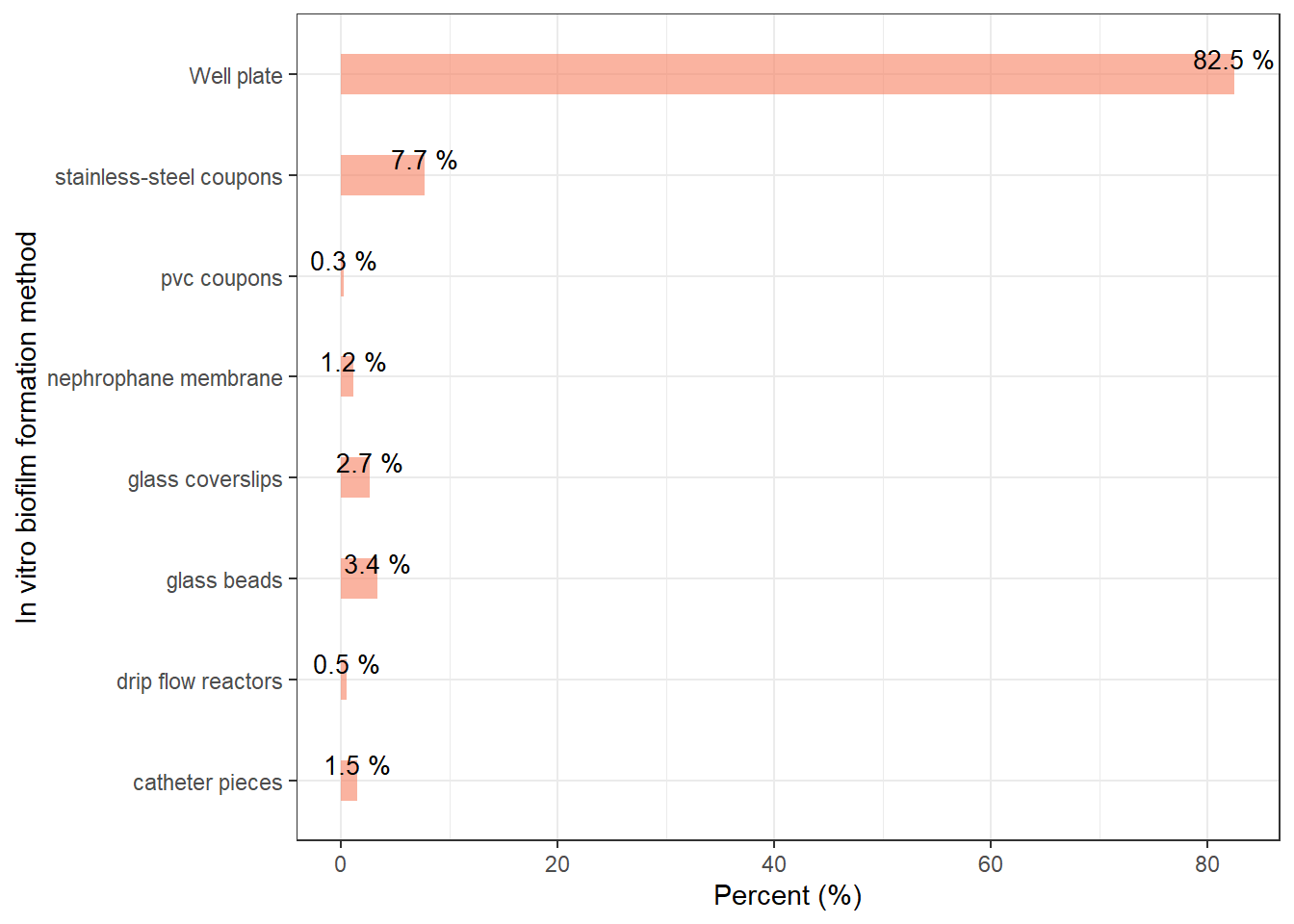


**Fig. S1.** Bacterial species used for biofilm formation. **Fig. S2.** In vitro biofilm formation surface.


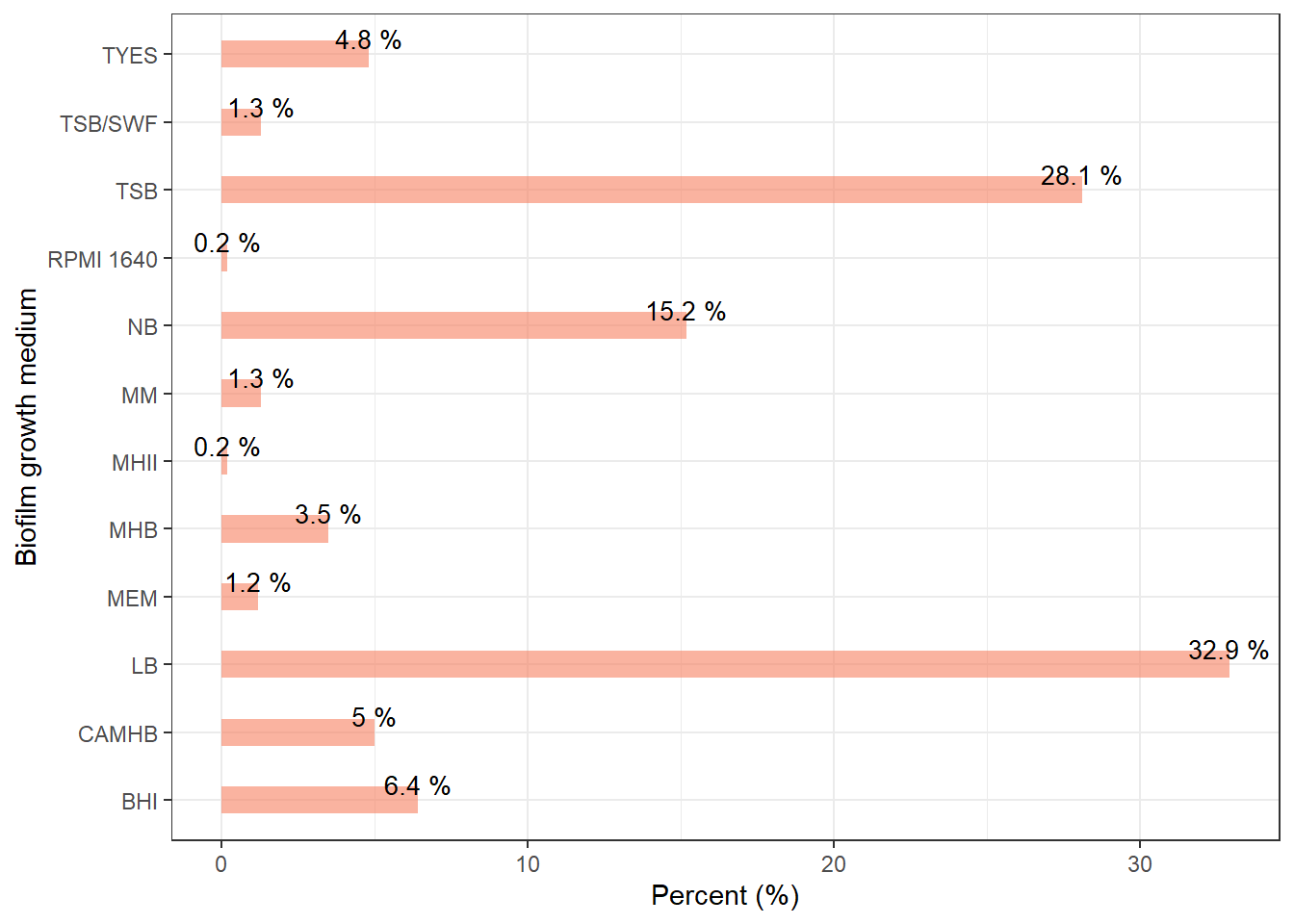

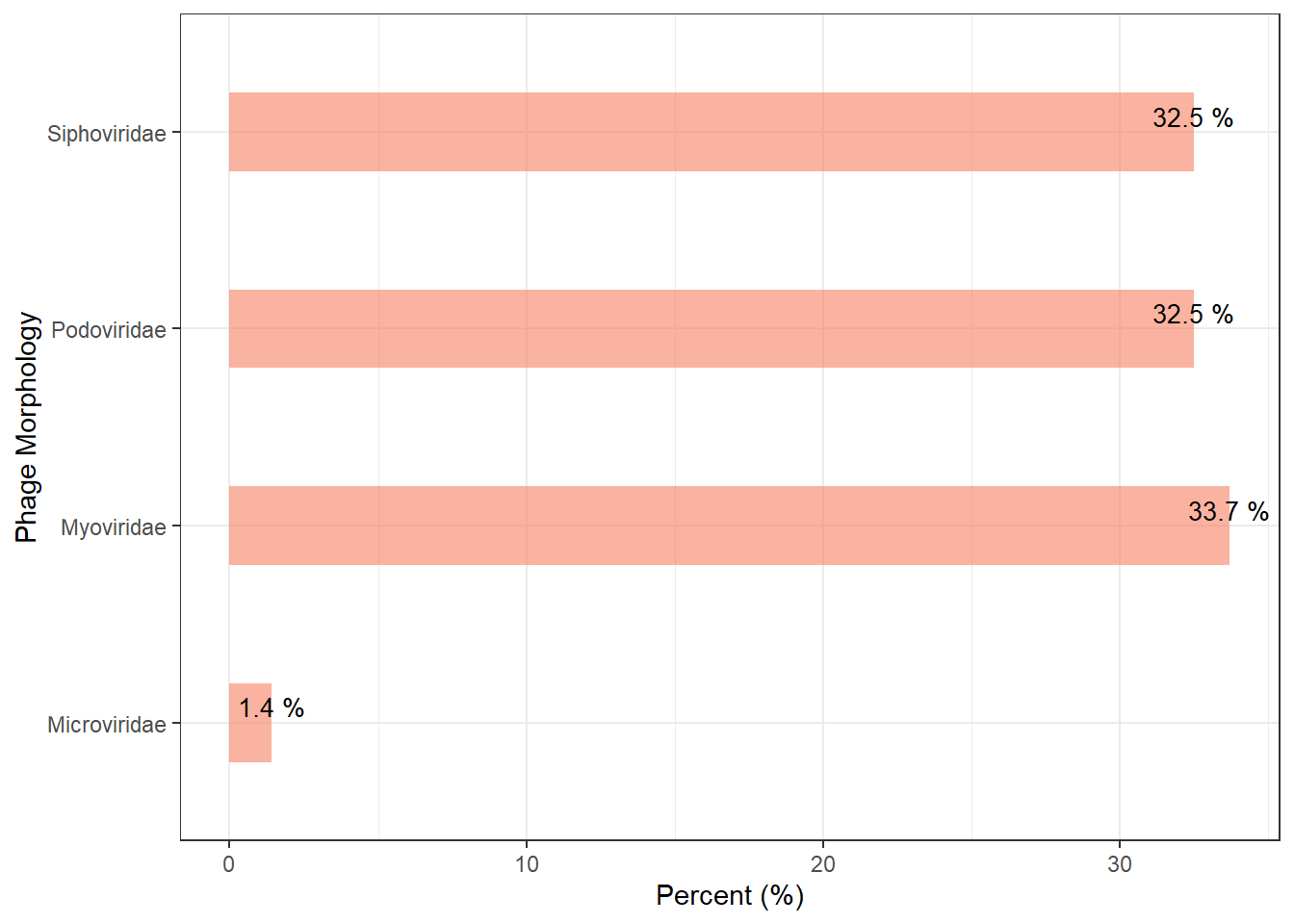


Siphovirus

Podovirus

Myovirus

Untailed

**Fig. S3.** Biofilm growth medium. **Fig. S4.** Morphology of the phages used for biofilm control.


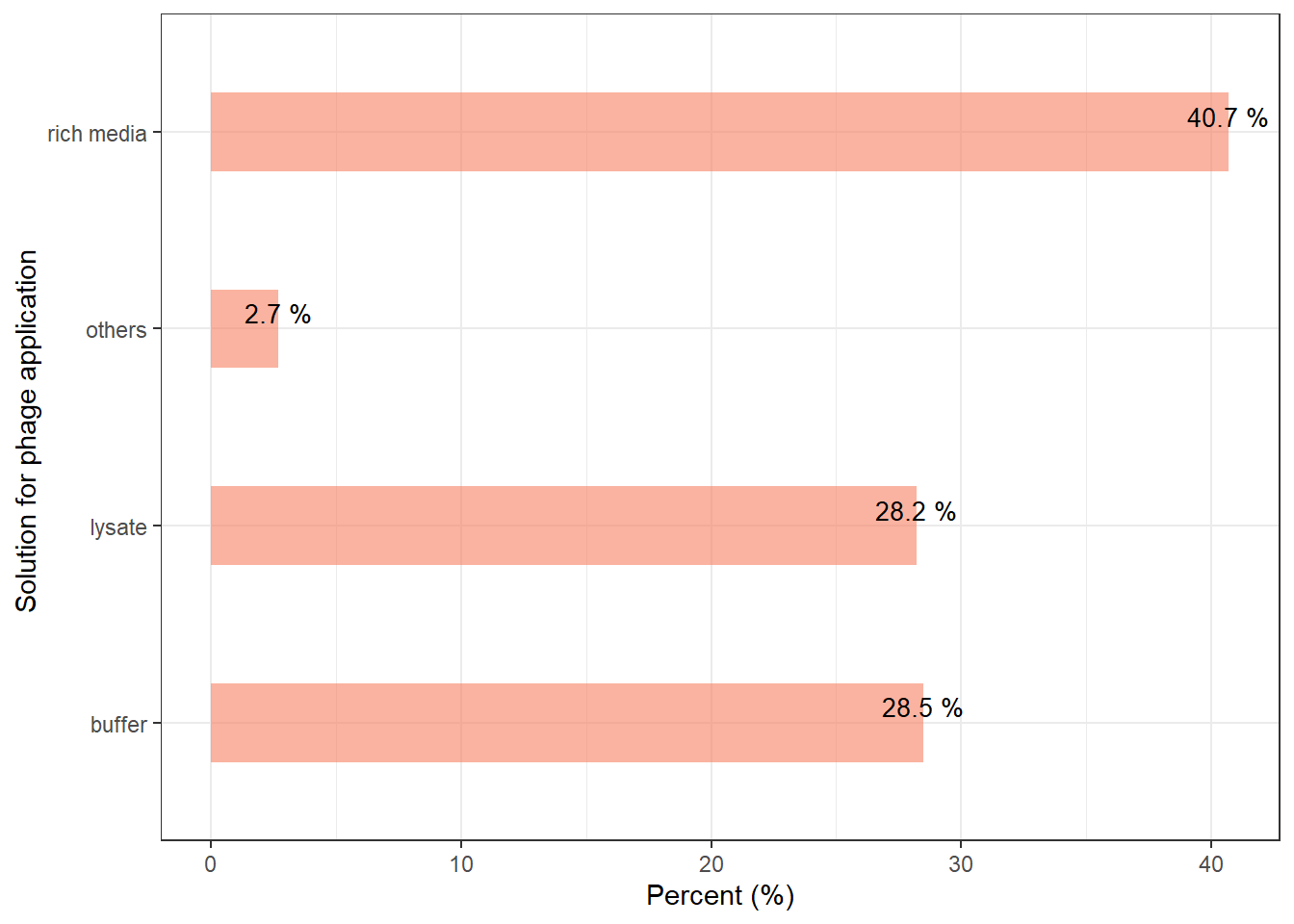

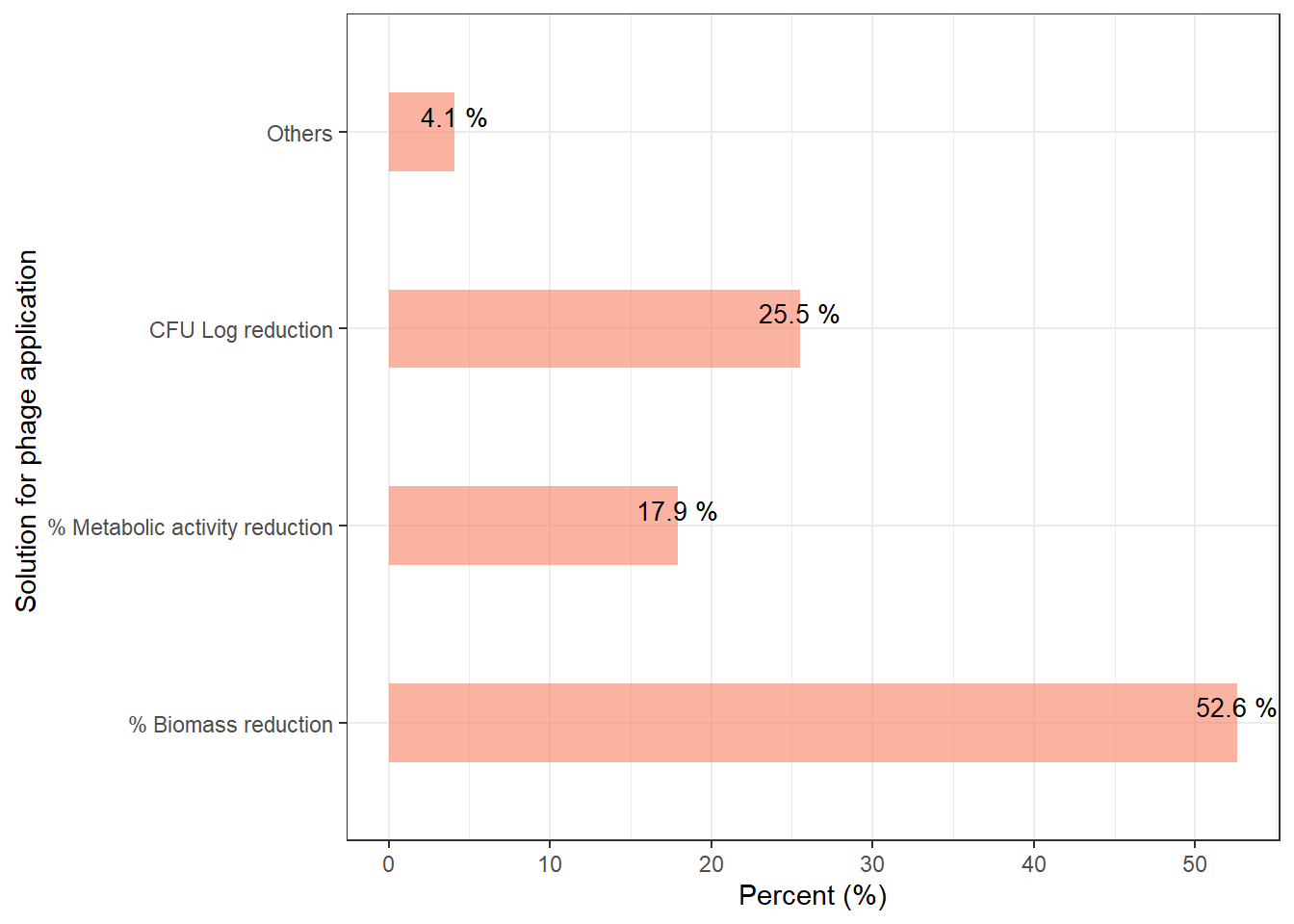


**Fig. S5.** Solution for phage application. **Fig. S6.** Biofilm assessment method.

b)

c)

a)

**
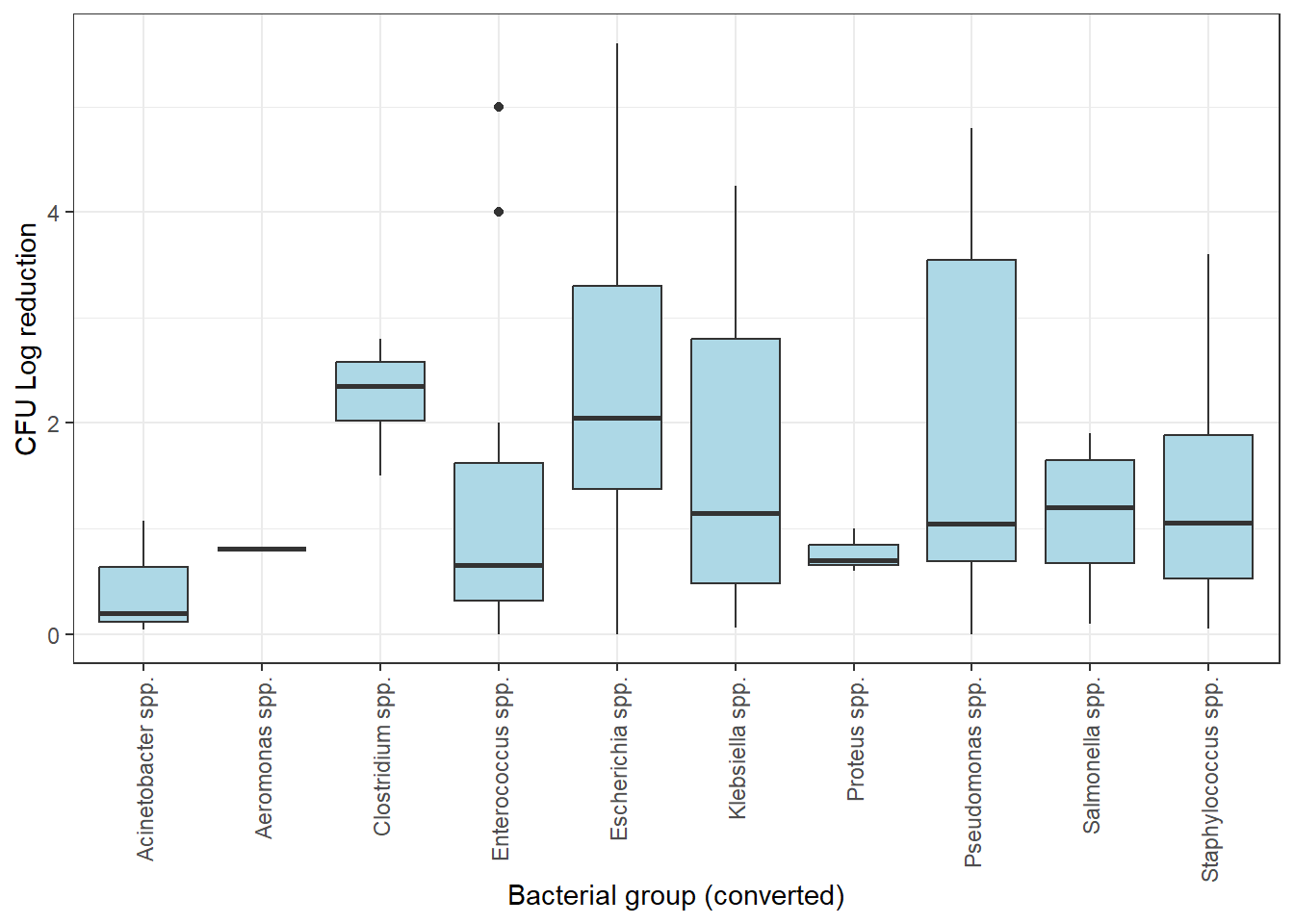

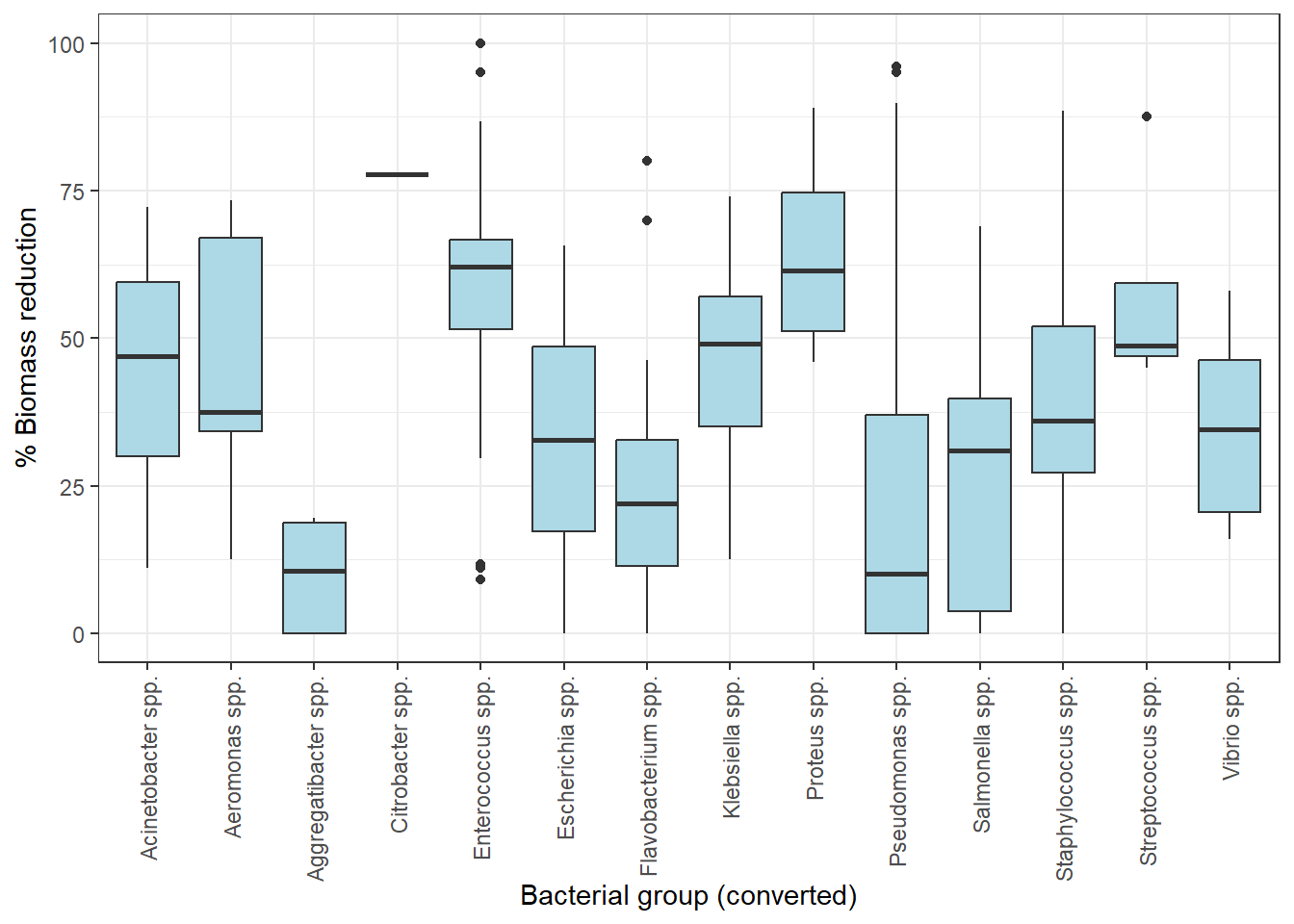

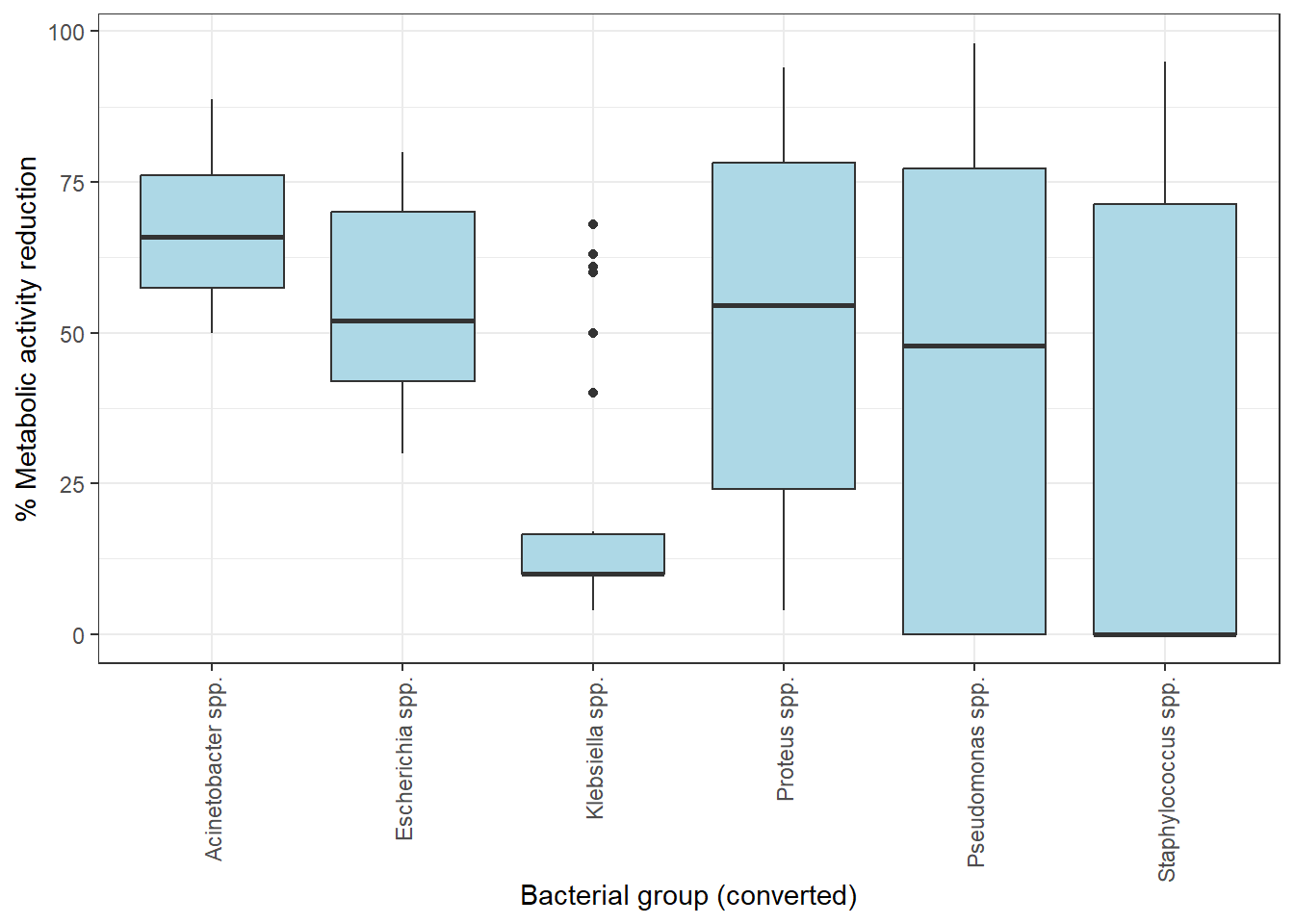
**

**Fig. S7.** Comparative analysis between bacterial species and: CFU log reduction (a), percentage of biomass reduction (b), and percentage of metabolic activity reduction (c).

**
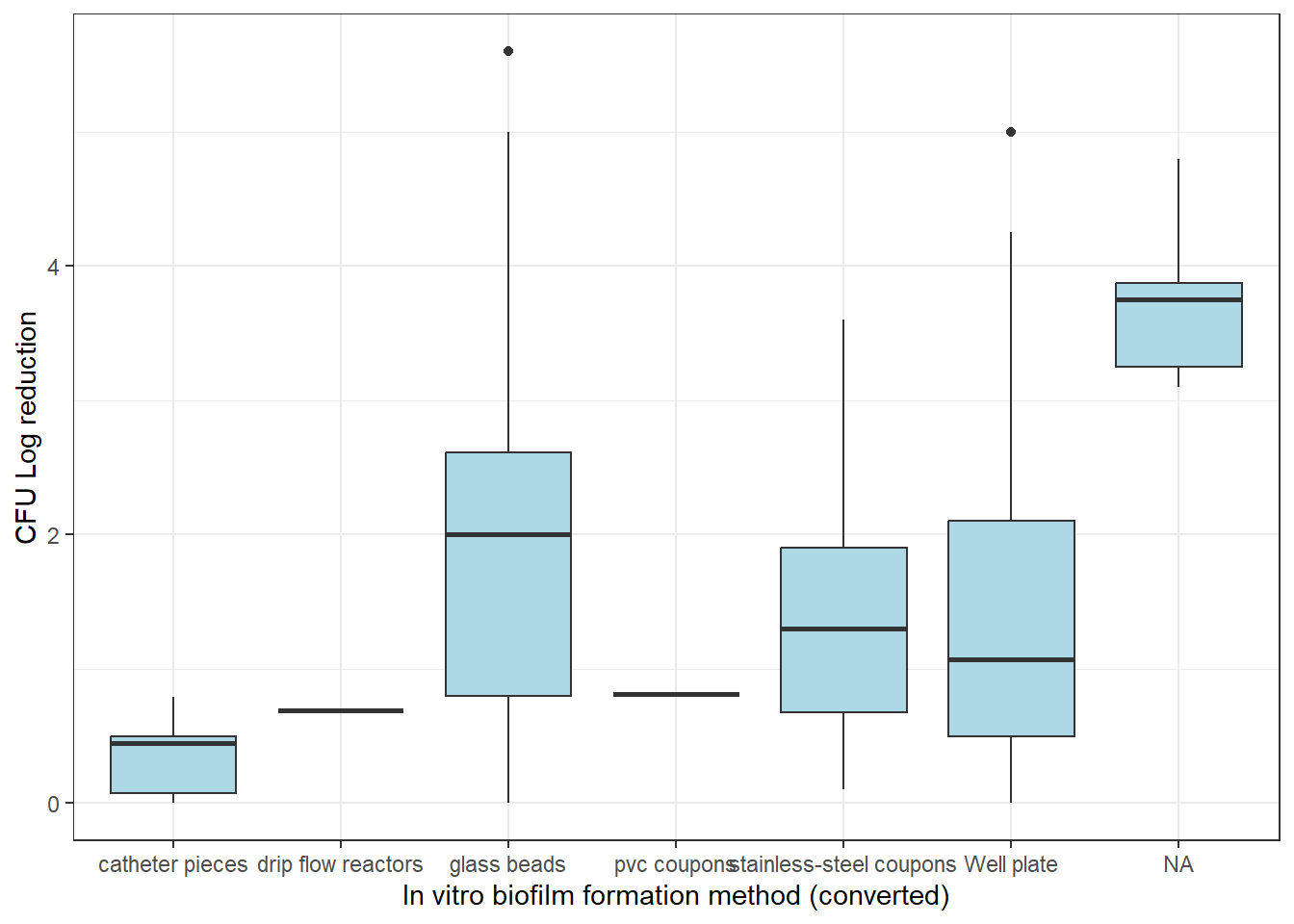

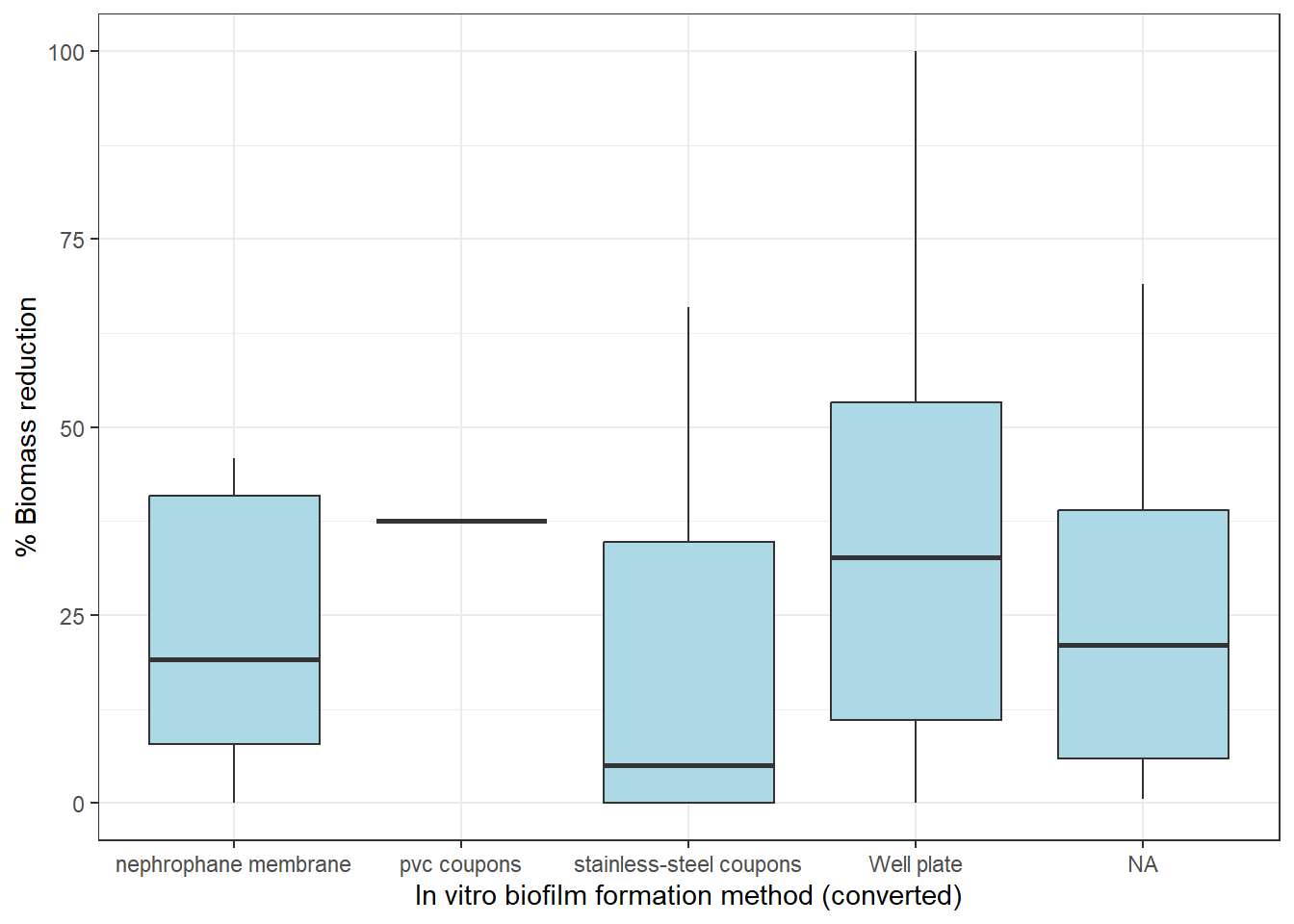

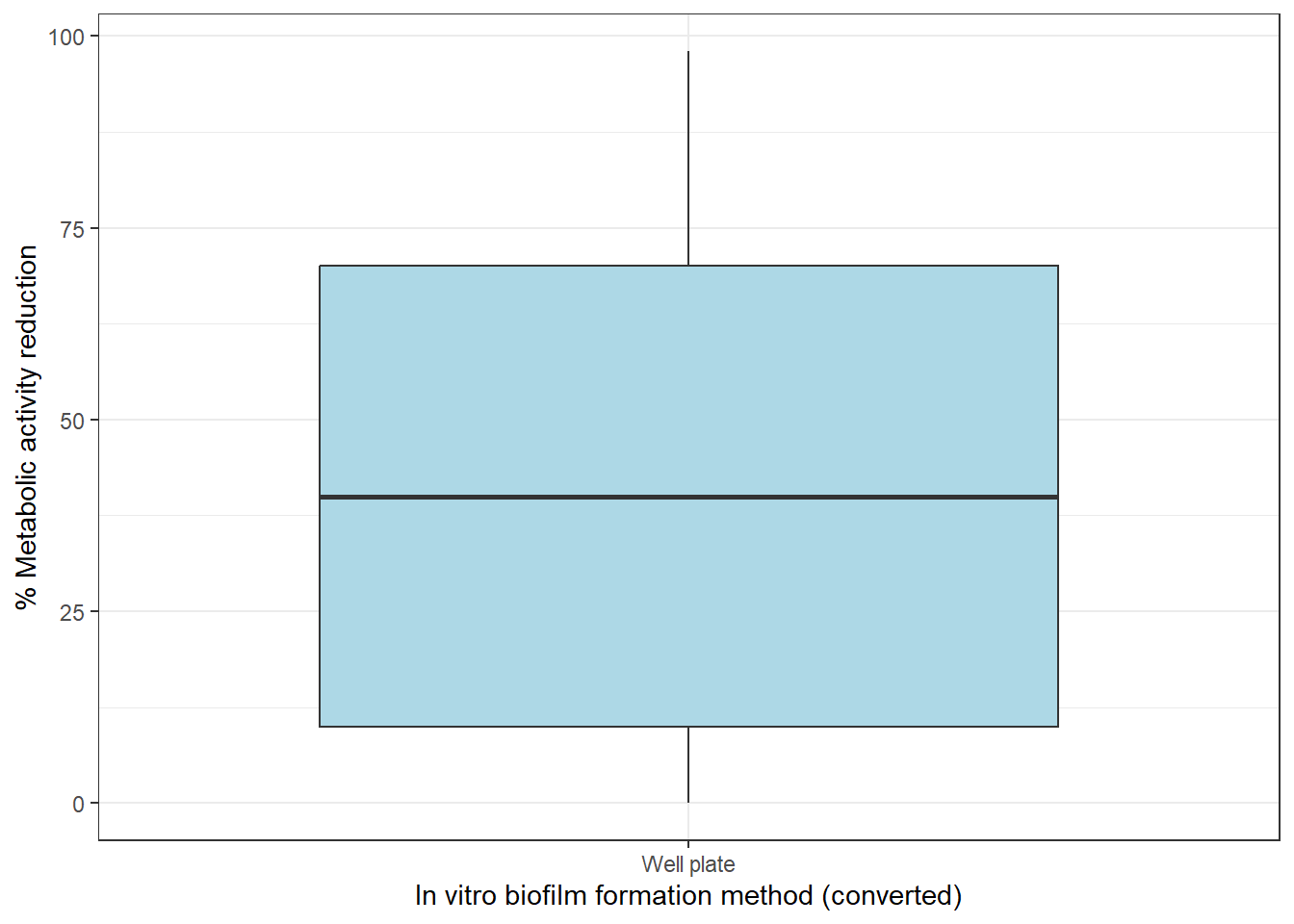
**

a)

b)

c)

**Fig. S8.** Comparative analysis between *in vitro* biofilm formation method and: CFU log reduction (a), percentage of biomass reduction (b), and percentage of metabolic activity reduction (c).

**
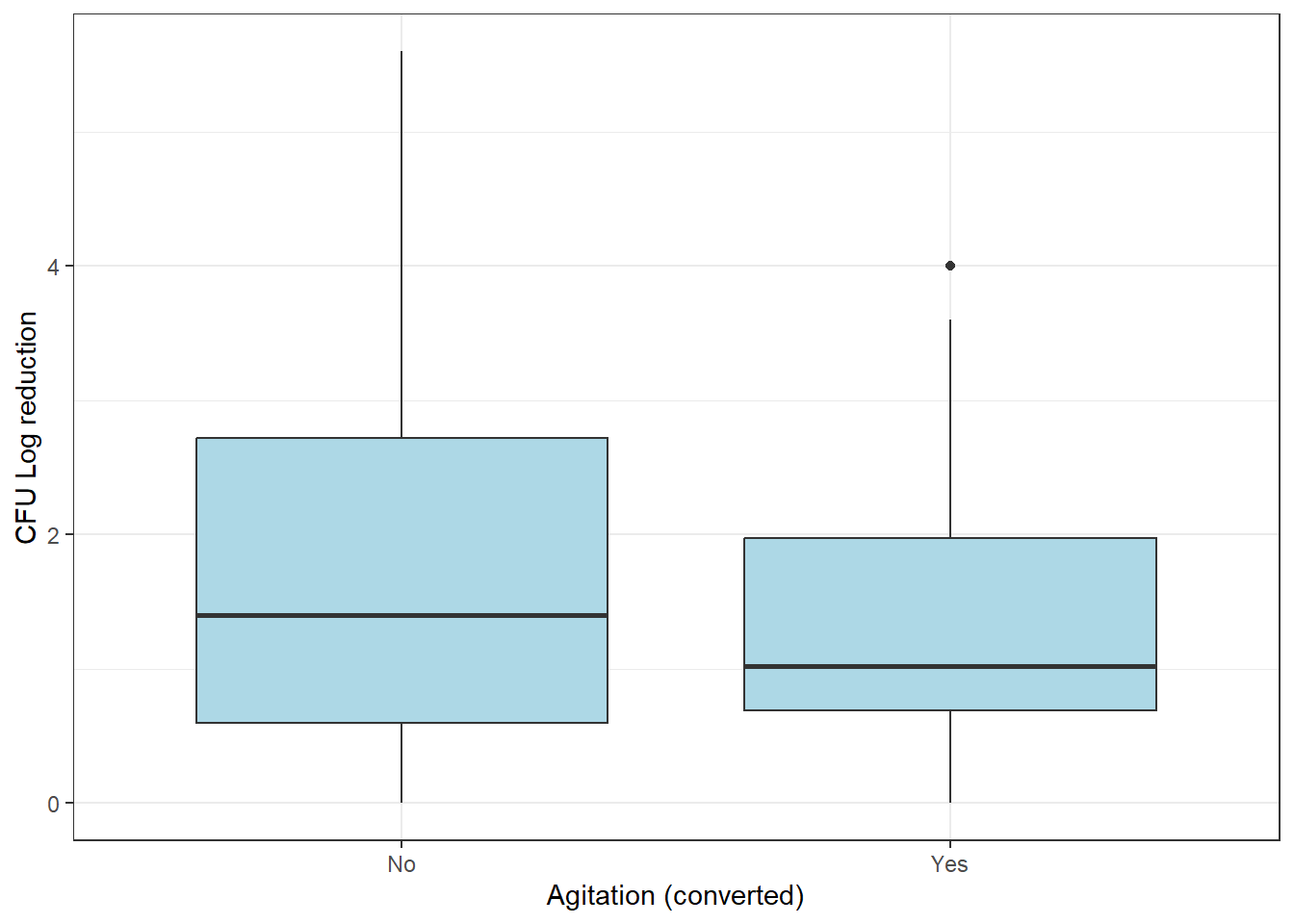

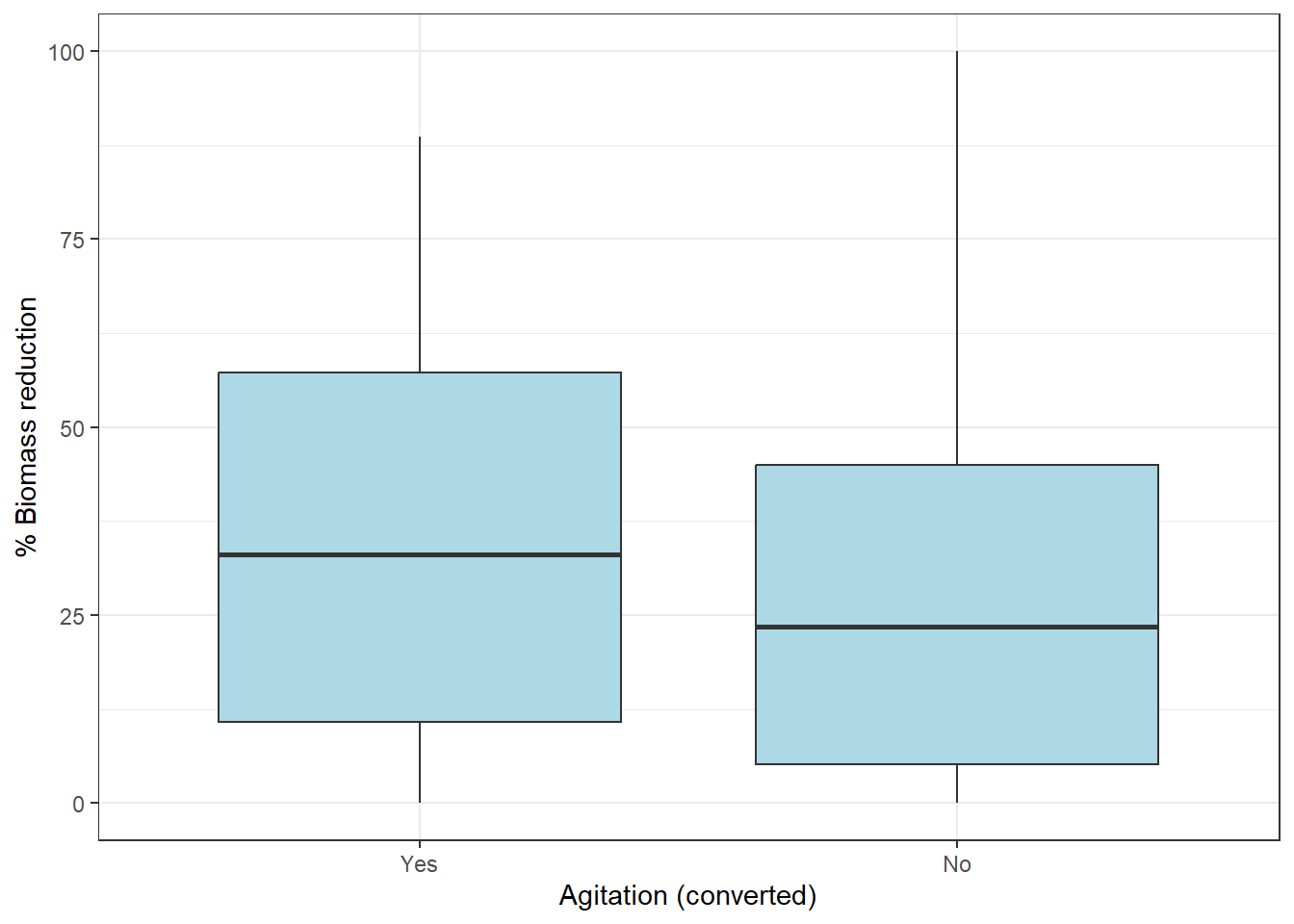

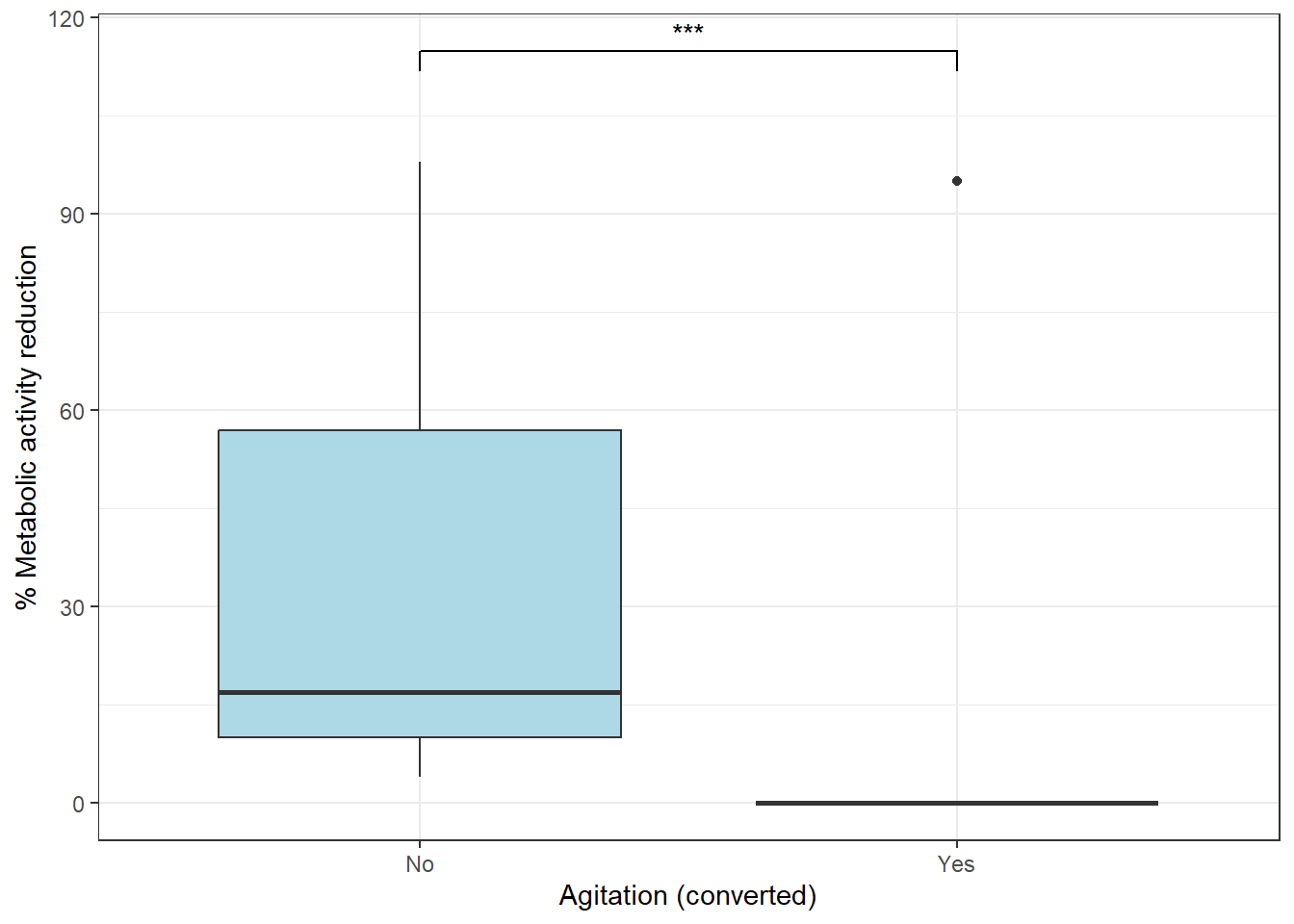
**

a)

b)

c)

**Fig. S9.** Comparative analysis between the use of agitation and: CFU log reduction (a), percentage of biomass reduction (b), and percentage of metabolic activity reduction (c). Statistically significant difference between the variables is marked with *** for p < 0.001.

a)

b)

c)

**
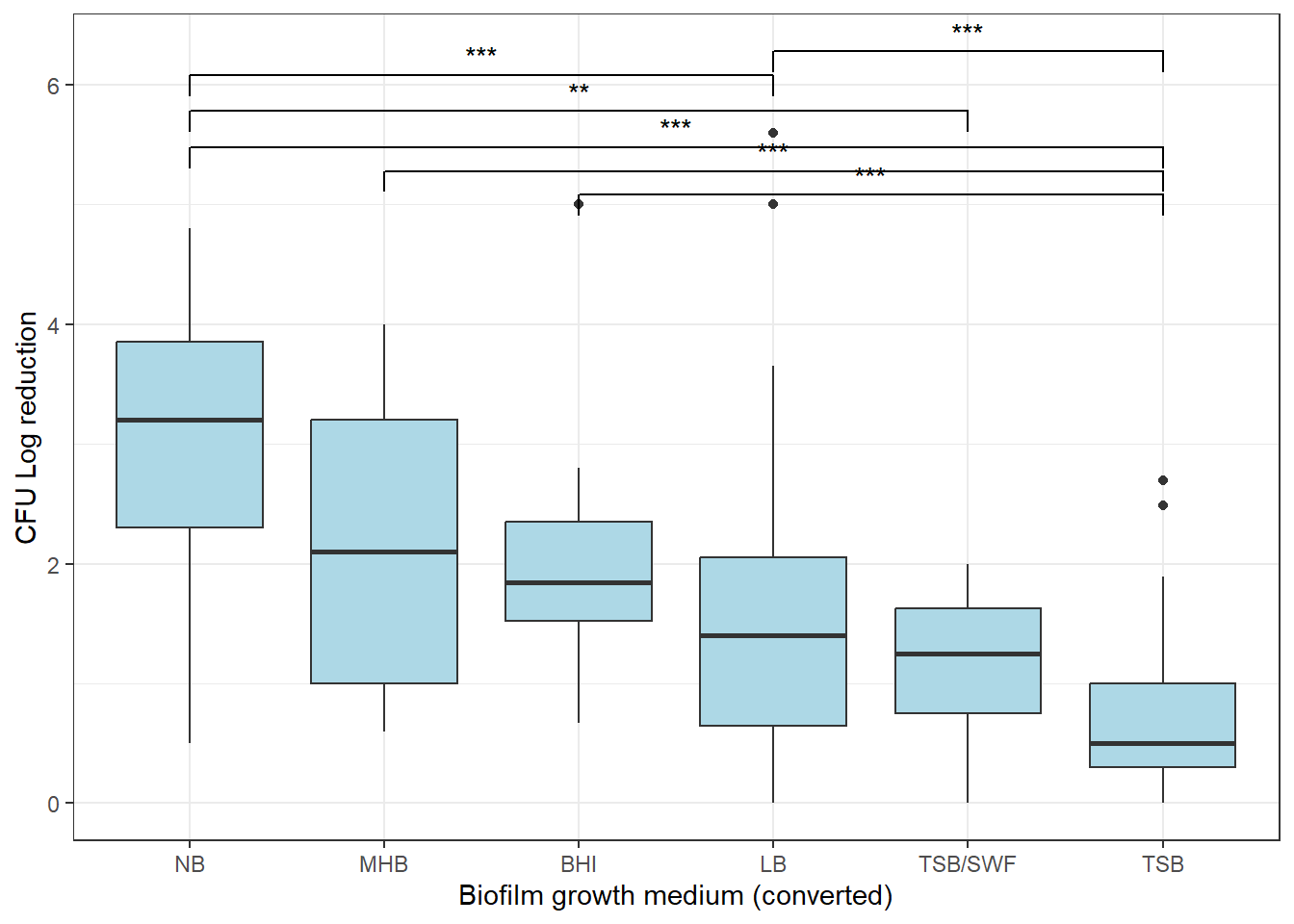

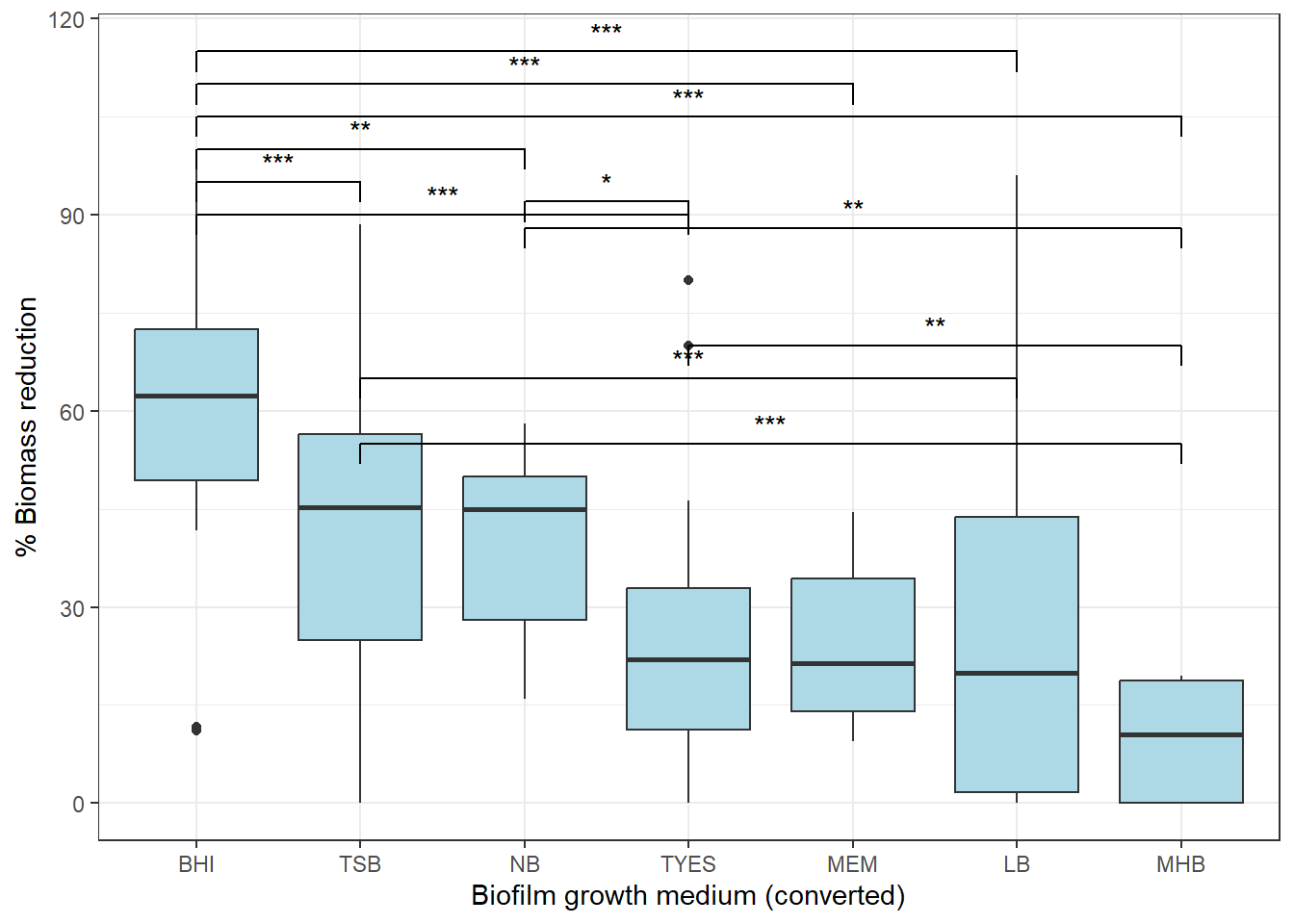

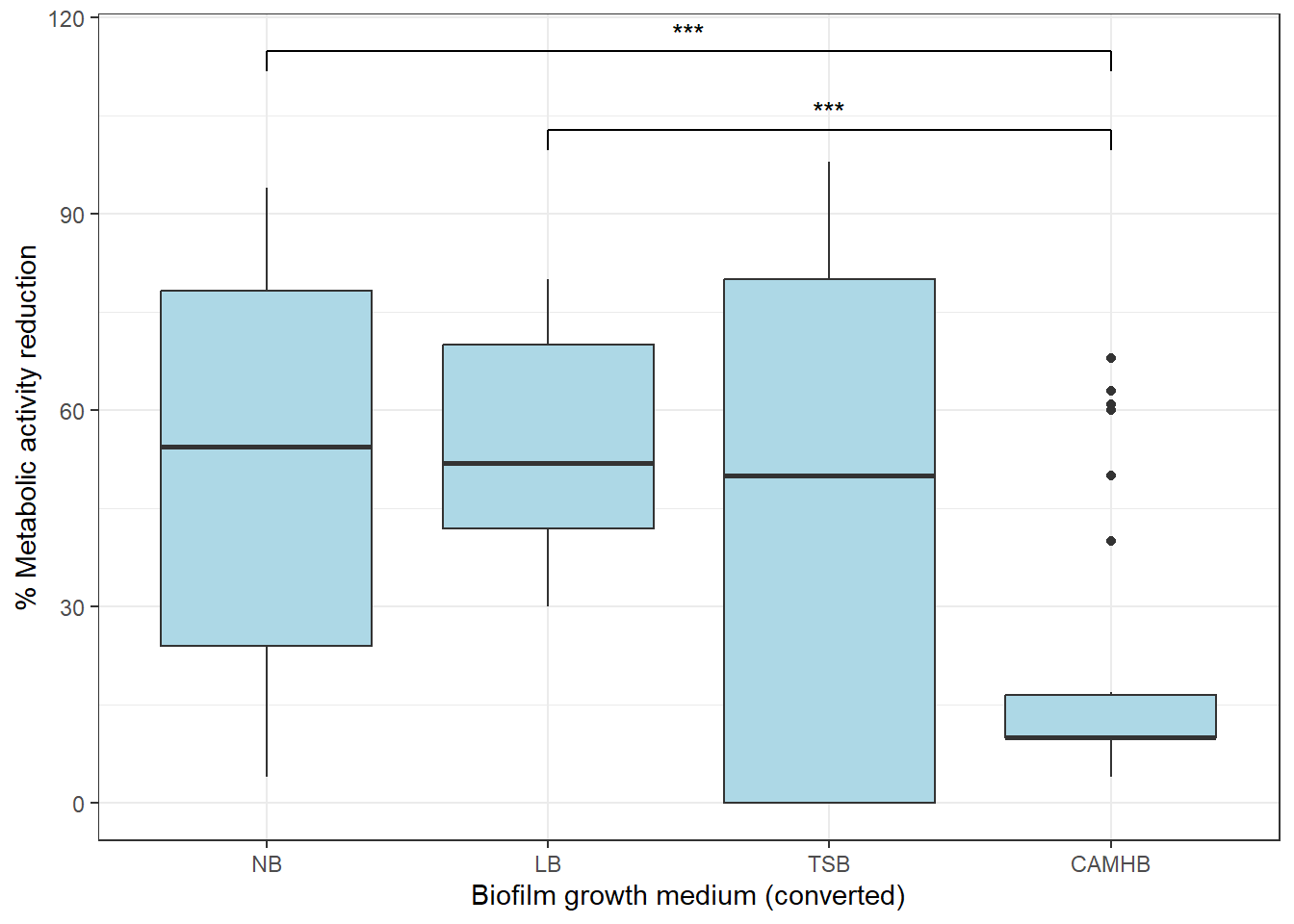
**

**Fig. S10.** Comparative analysis between biofilm growth medium and: CFU log reduction (a), percentage of biomass reduction (b), and percentage of metabolic activity reduction (c). Statistically significant differences between the variables are marked with * for p < 0.05, ** p < 0.01, and *** for p < 0.001.

c)

b)

a)

**
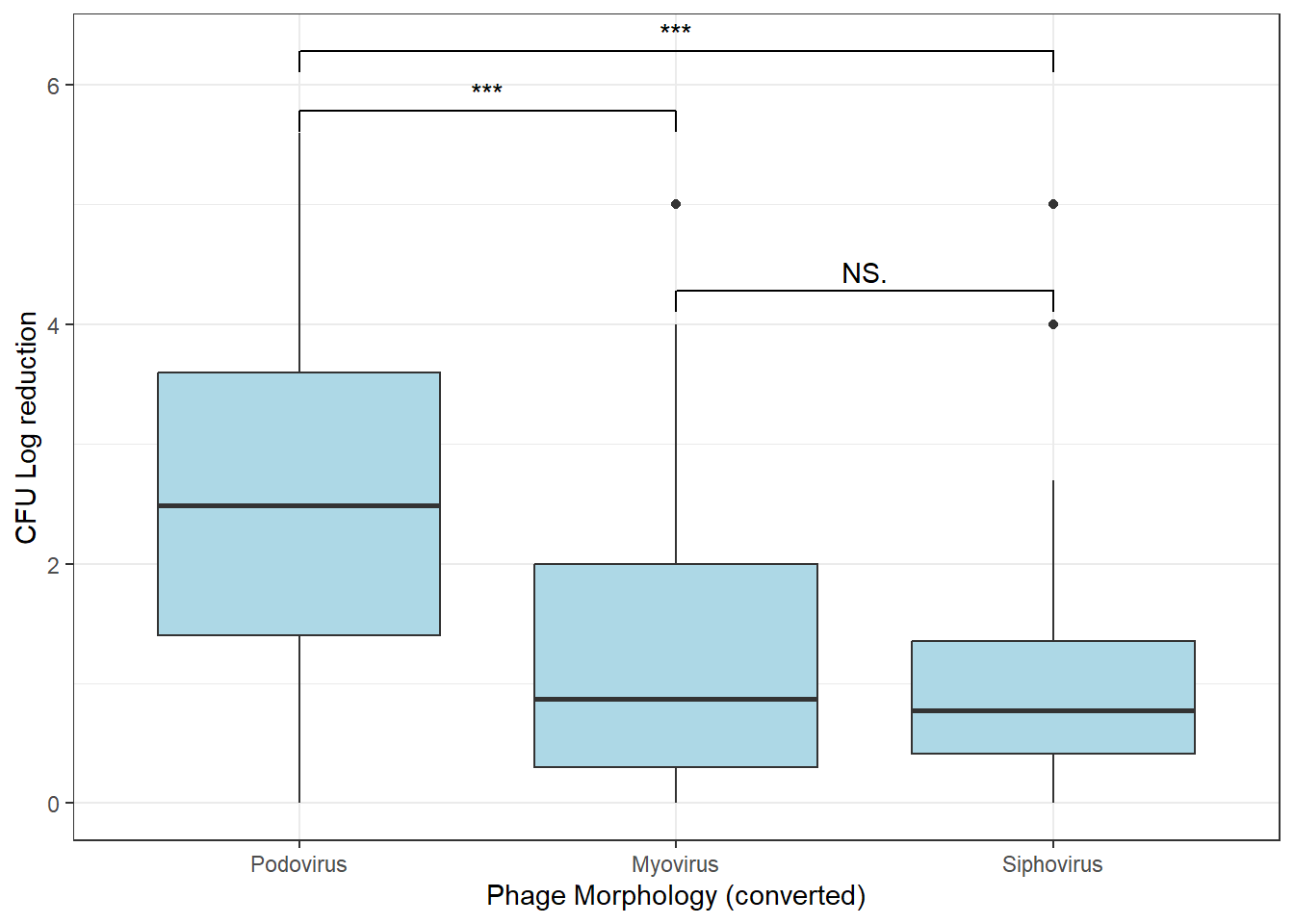

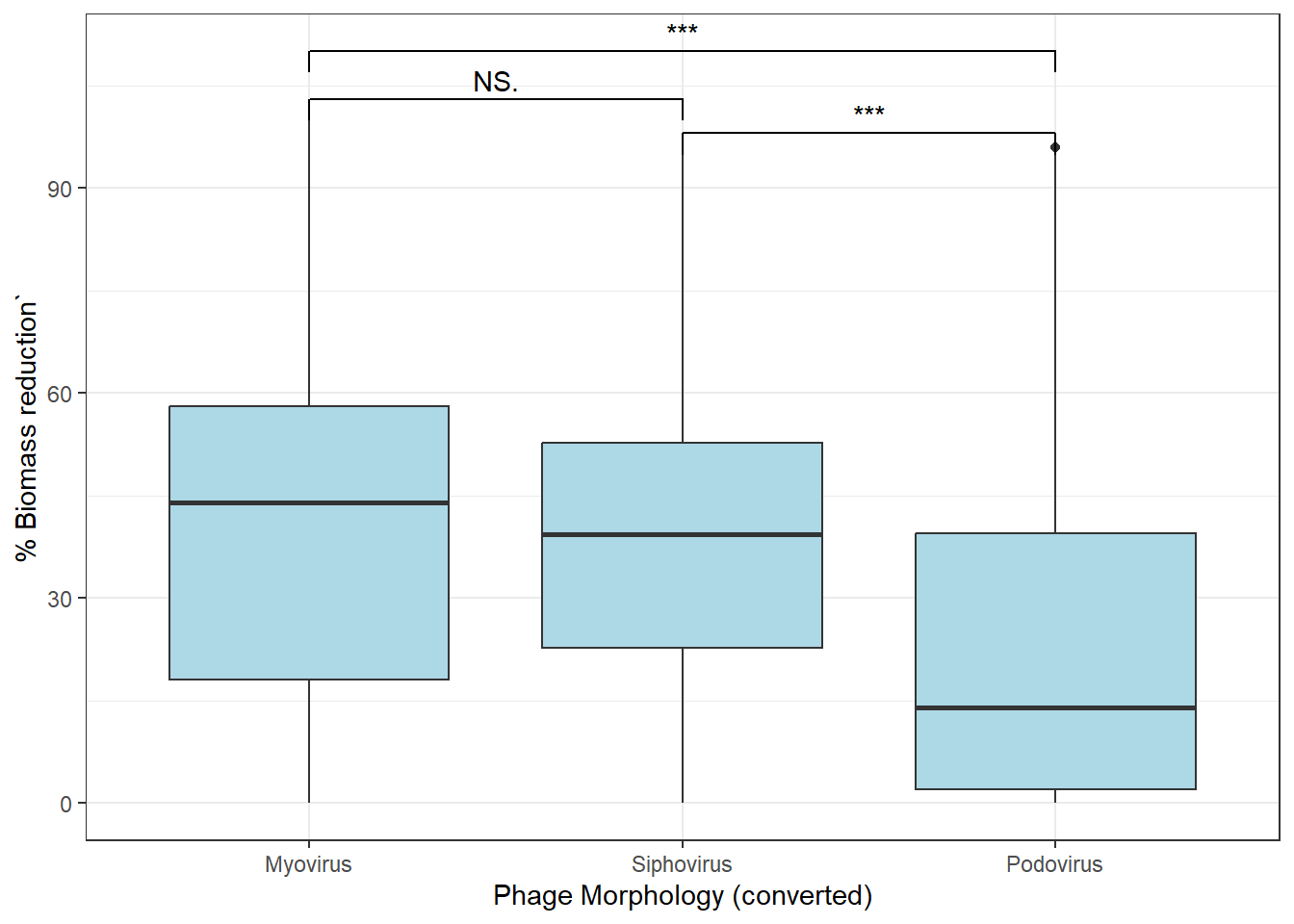

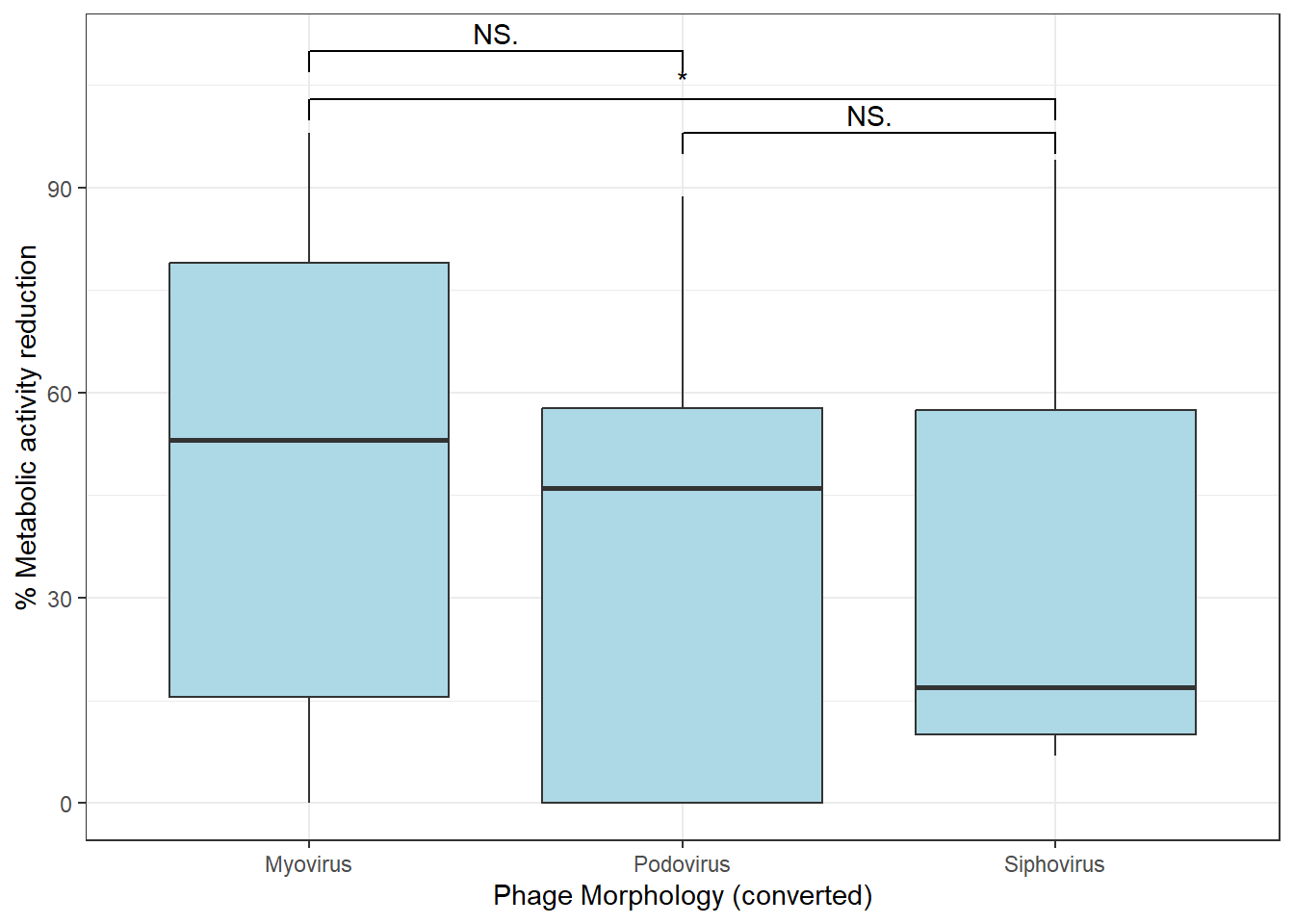
**

**Fig. S11.** Comparative analysis between phage morphology and: CFU log reduction (a), percentage of biomass reduction (b), and percentage of metabolic activity reduction (c). Statistically significant differences between the variables are marked with *** for p < 0.001 and NS when no statistically significant difference was found.

**
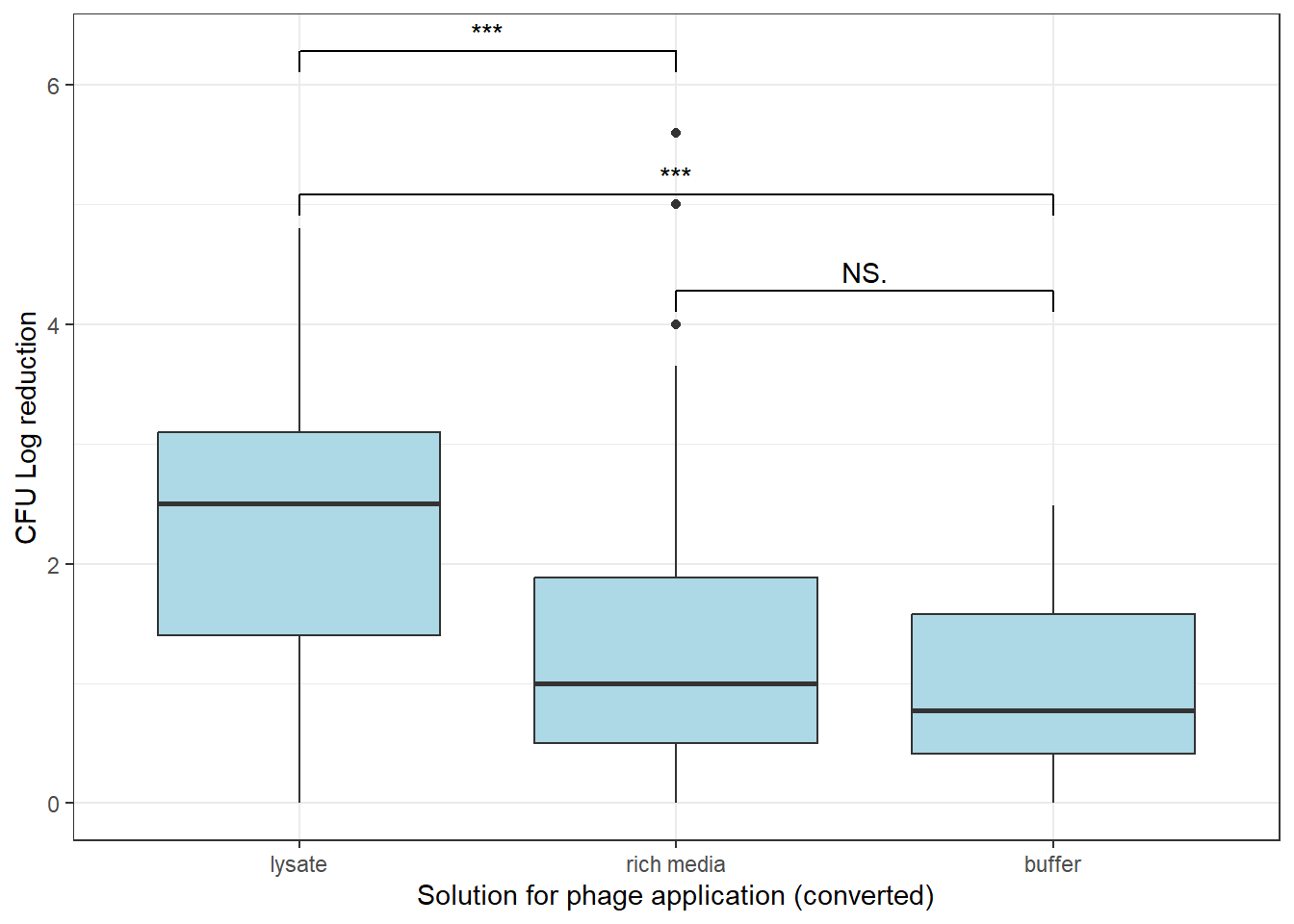

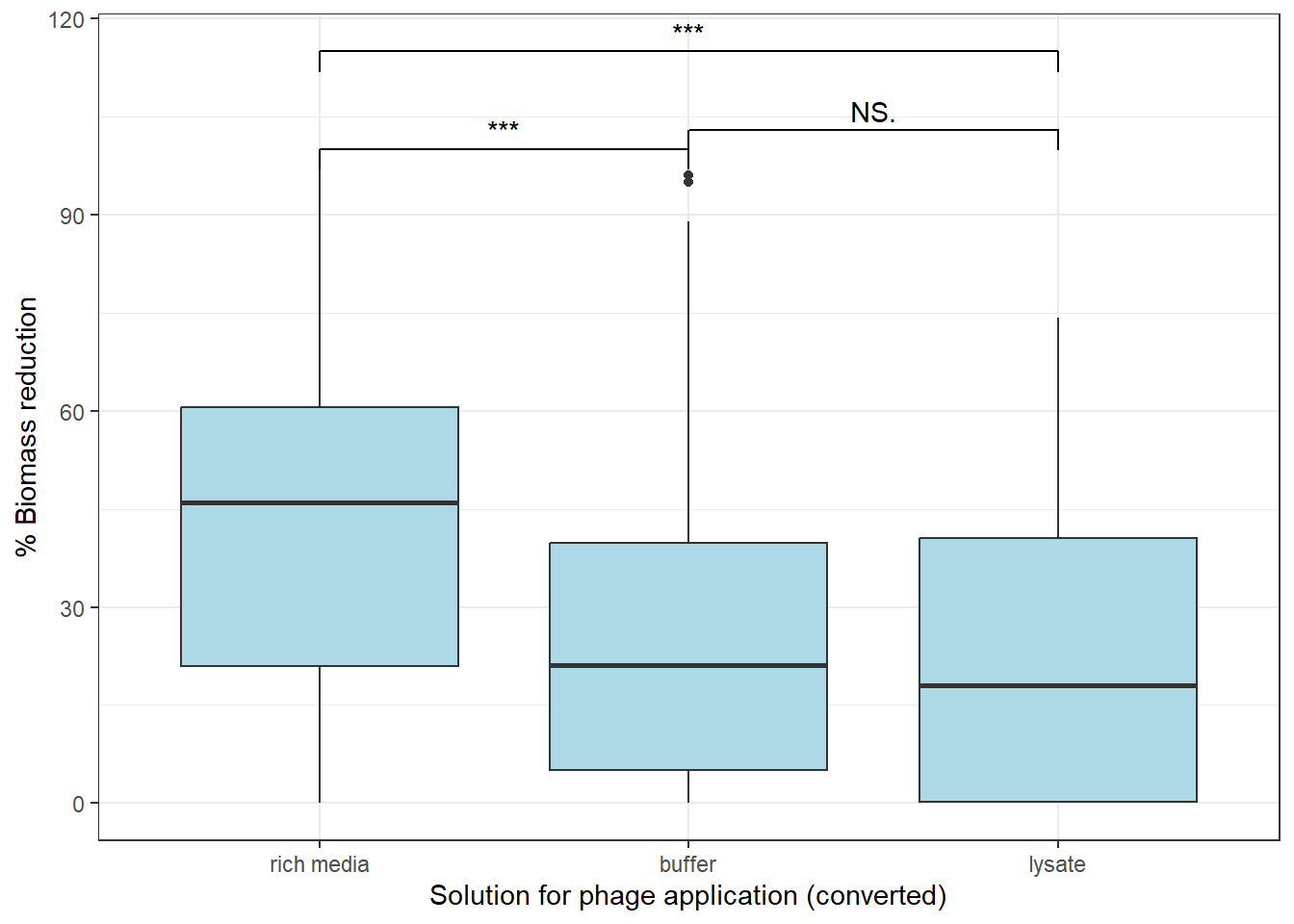

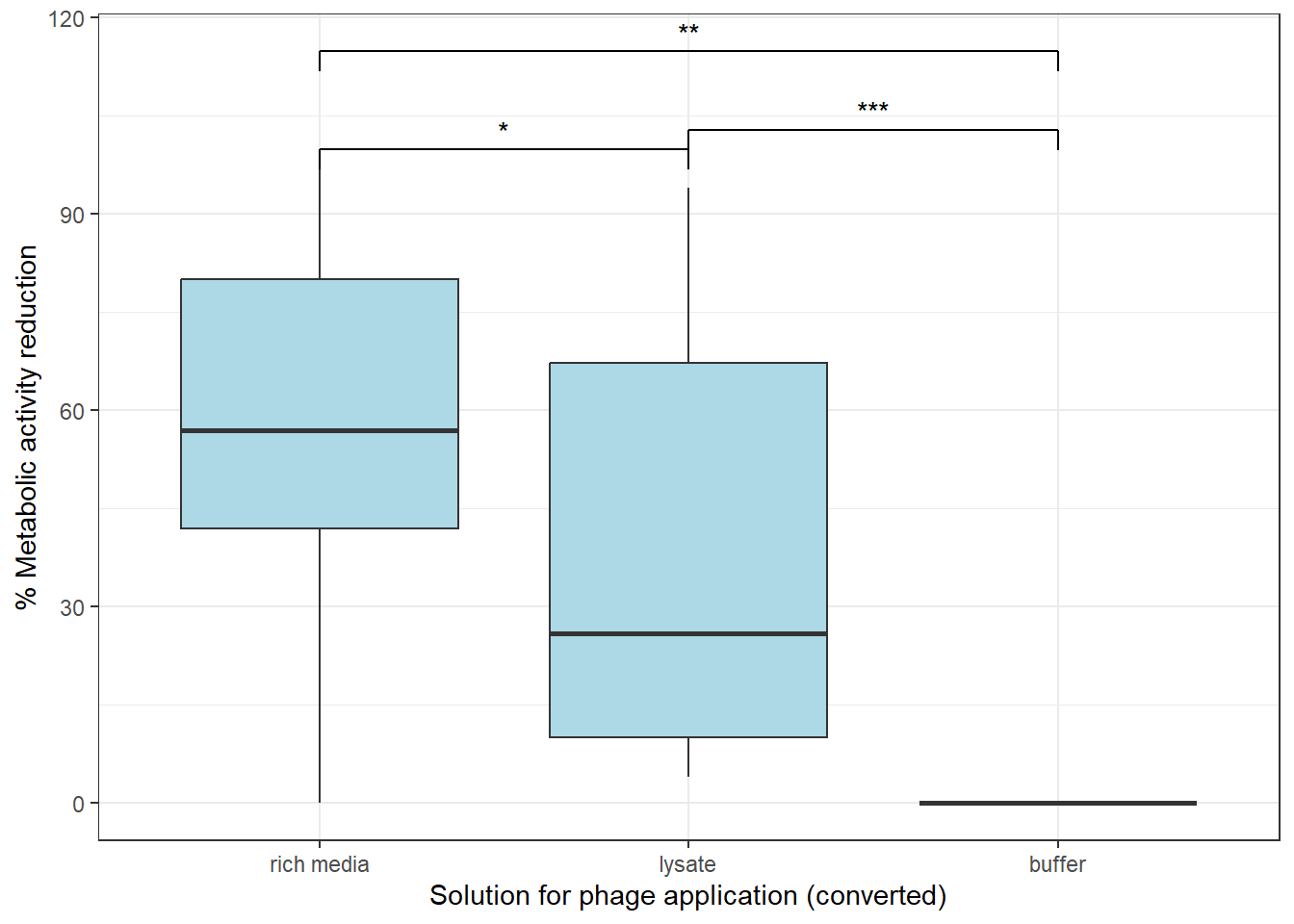
**

c)

b)

a)

**Fig. S12.** Comparative analysis between the solution used for phage application and: CFU log reduction (a), percentage of biomass reduction (b), and percentage of metabolic activity reduction (c). Statistically significant differences between phage application solution are marked with * for p < 0.05, ** p < 0.01, *** for p < 0.001, and NS when no statistically significant difference was found.
